# Cervicovaginal lavages uncover growth factors as key biomarkers for early diagnosis and prognosis of endometrial cancer

**DOI:** 10.1186/s43556-024-00219-6

**Published:** 2024-11-08

**Authors:** Hannah J. Harris, Paweł Łaniewski, Haiyan Cui, Denise J. Roe, Dana M. Chase, Melissa M. Herbst-Kralovetz

**Affiliations:** 1 Department of Obstetrics and Gynecology, College of Medicine-Phoenix, University of Arizona, Phoenix, AZ USA; 2 Department of Life Sciences, University of Bath, Bath, UK; 3 Department of Basic Medical Sciences, College of Medicine-Phoenix, University of Arizona, Phoenix, AZ USA; 4 University of Arizona Cancer Center, Tucson, AZ USA; 5 Department of Epidemiology and Biostatistics, Mel and Enid Zuckerman College of Public Health, University of Arizona, Tucson, AZ USA; 6 Division of Gynecologic Oncology, Department of Obstetrics and Gynecology, David Geffen School of Medicine, University of California Los Angeles, Los Angeles, CA USA

**Keywords:** Endometrial cancer, Protein biomarkers, Cervicovaginal microenvironment, Tumor characteristics

## Abstract

Endometrial cancer (EC) rates are continuing to rise and it remains the most common gynecologic cancer in the US. Existing diagnostic methods are invasive and can cause pain and anxiety. Hence, there is a need for less invasive diagnostics for early EC detection. The study objective was to evaluate the utility of growth factors collected through minimally invasive cervicovaginal lavage (CVL) sampling as diagnostic and prognostic biomarkers for EC. CVL samples from 192 individuals undergoing hysterectomy for benign or malignant conditions were collected and used to quantify the concentrations of 19 growth and angiogenic factors using multiplex immunoassays. Patients were categorized based on disease groups: benign conditions (*n* = 108), endometrial hyperplasia (*n* = 18), and EC (*n* = 66). EC group was stratified into grade 1/2 endometrial endometrioid cancer (*n* = 53) and other EC subtypes (*n* = 13). Statistical associations were assessed using receiver operating characteristics, Spearman correlations and hierarchical clustering. Growth and angiogenic factors: angiopoietin-2, endoglin, fibroblast activation protein (FAP), melanoma inhibitory activity, and vascular endothelial growth factor-A (VEGF-A) were significantly (*p* < 0.0001) elevated in EC patients. A multivariate model combining 11 proteins with patient age and body mass index exhibited excellent discriminatory potential (area under curve = 0.918) for EC, with a specificity of 90.7% and a sensitivity of 87.8%. Moreover, angiopoietin-2, FAP and VEGF-A significantly (*p* < 0.05–0.001) associated with tumor grade, size, myometrial invasion, and mismatch repair status. Our results highlight the innovative use of growth and angiogenic factors collected through CVL sampling for the detecting endometrial cancer, showcasing not only their diagnostic potential but also their prognostic value.

**Supplementary Information:**

The online version contains supplementary material available at 10.1186/s43556-024-00219-6.

## Introduction

Endometrial cancer (EC) is the fourth most prevalent cancer among individuals with female reproductive organs. In the United States, there will be an estimated 67,880 new cases of EC in 2024 alone [1]. EC incidence has been increasing by approximately 1% per year since the mid-2000s; worryingly, at the same time, the incidence rate for Black, Hispanic, Asian American, and Pacific Islander females has been increasing by over 2% per year [1]. Compounding this issue, Black, Hispanic, and Native American females are often diagnosed with more advanced stages of EC, and thus have a higher mortality rate compared to non-Hispanic White females [2–4]. While traditionally viewed as affecting older, postmenopausal females, the incidence of EC among individuals under 40 years old is on the rise. For instance, between 2000 and 2017, the incidence rate among females aged 30–34 years old has risen from 0.7 to 2.0 per 100,000, and for those aged 35–39 years old, the rate has increased from 2.2 to 4.0 per 100,000 [5]. Given this upward trend, there is an increasing need for early EC detection tools.

Understanding the risk factors associated with EC is imperative for developing early detection tools. Risk factors for EC include increased age, post-menopause, diabetes, obesity, nulliparity, unopposed estrogen therapy, and tamoxifen use [6, 7]. Traditionally, EC was categorized into two types: type 1 EC which consisted of grade 1 or 2 endometrioid tumors, estrogen-driven and the most common type, and more aggressive type 2 EC which consisted of higher-grade tumors that had a less favorable prognosis and were less common than type 1 [7]. The Cancer Genome Atlas Project recently identified four distinct molecular groups across EC based on copy number alterations and mutation load [8]. The subgroups identified were as follows: mismatch repair deficiency (MMRd), hypermutated polymerase epsilon (*POLE*mut), abnormal p53, and no specific mutation profile (NSMP). Identifying these four molecular types allows for a more accurate understanding of tumor characteristics, clinical prognosis and guides treatment. The overall prognosis and risk stratification of EC is determined based on numerous factors, including tumor characteristics, such as histological grade, tumor size, and presence of myometrial and lymphovascular invasion [9]. Myometrial and lymphovascular invasion are well-recognized as predictors of disease outcomes [10]. For example, deep myometrial invasion (> 50% invasion of the myometrium) has been associated with a poor survival rate and recurrence in EC [11]. Research has shown that tumor size also has a prognostic value [12], and tumors larger than 2 cm have been associated with EC recurrence [13–15].

EC often manifests with abnormal uterine bleeding (AUB) or postmenopausal bleeding (PMB), and patients with more advanced disease may present with pelvic or abdominal pain [16]. The most common symptom of EC is AUB or PMB, but AUB and PMB are common symptoms of other gynecologic conditions, such as adenomyosis, endometrial polyps, fibroids and polycystic ovarian syndrome [17]. Recognizing the symptoms early is vital for timely diagnosis and intervention in EC. Currently, diagnosis relies on a series of painful procedures that could act as barriers to diagnosis, as they are known to cause pain and discomfort and can be anxiety-inducing [18]. At present, symptomatic females first undergo a transvaginal ultrasound (TVUS) [19], which is utilized to measure the width of the endometrium. A TVUS has a negative predictive value of 99% [20] for EC, although a relatively low positive predictive value for EC [6]. Thus, more invasive procedures are required. Furthermore, TVUS is less specific in premenopausal females [21], and in a simulated cohort, TVUS missed four times as many cases of EC in Black females, compared to White females [22]. Moreover, obesity and diabetes are known to decrease the accuracy of TVUS [23]. After an abnormal TVUS result, a patient must undergo further procedures for an accurate diagnosis. A dilation and curettage or an endometrial biopsy with or without hysteroscopy are commonly used. Yet, research has shown that both endometrial biopsy and dilation and curettage cause pain and anxiety in patients [24, 25].

There is a need for less painful and invasive diagnostic methods in EC. Previous biomarker research in EC has focused on serum biomarkers for EC detection. The two that exhibited the most promise were cancer antigen (CA) 125 and human epididymis (HE) 4. Serum CA125 and HE4 are elevated in EC, however, further research has exhibited that these markers display low sensitivity for EC [26, 27]. This could be due to the relatively low concentration in peripheral blood samples [28]. Previously, Łaniewski et al. [29] demonstrated cervicovaginal lavage (CVL) shows promise as a minimally invasive sample collection technique for EC detection as CVLs allow for localized sampling of the lower reproductive tract due to the anatomical continuity of the female reproductive tract [30]. The continual rise of EC necessitates the development of innovative minimally invasive diagnostic methods that could enhance early detection, such as CVL sampling. CVL sampling in combination with a robust protein biomarker model represents an innovative opportunity that could be advanced to point of care testing.

Herein, by leveraging our previous study, in which we demonstrated CVLs as promising diagnostic sample collection method for EC [29], we hypothesized that specific markers of angiogenesis and growth factors, collected in CVLs and not previously tested, could be utilized to improve detection of EC and hold prognostic utility. Through the localized sampling provided by CVLs in combination with multiplex immune assays, we identified specific proteins and combinations thereof with diagnostic and prognostic value in EC. We utilized machine learning algorithms to enhance the overall predictive accuracy, sensitivity, and specificity of EC detection. Further, we used the lower reproductive tract sampling of CVLs and tumor characteristics to understand the prognostic utility of the proteins assessed.

## Results

### Patient demographics and characteristics

**Fig. 1:**
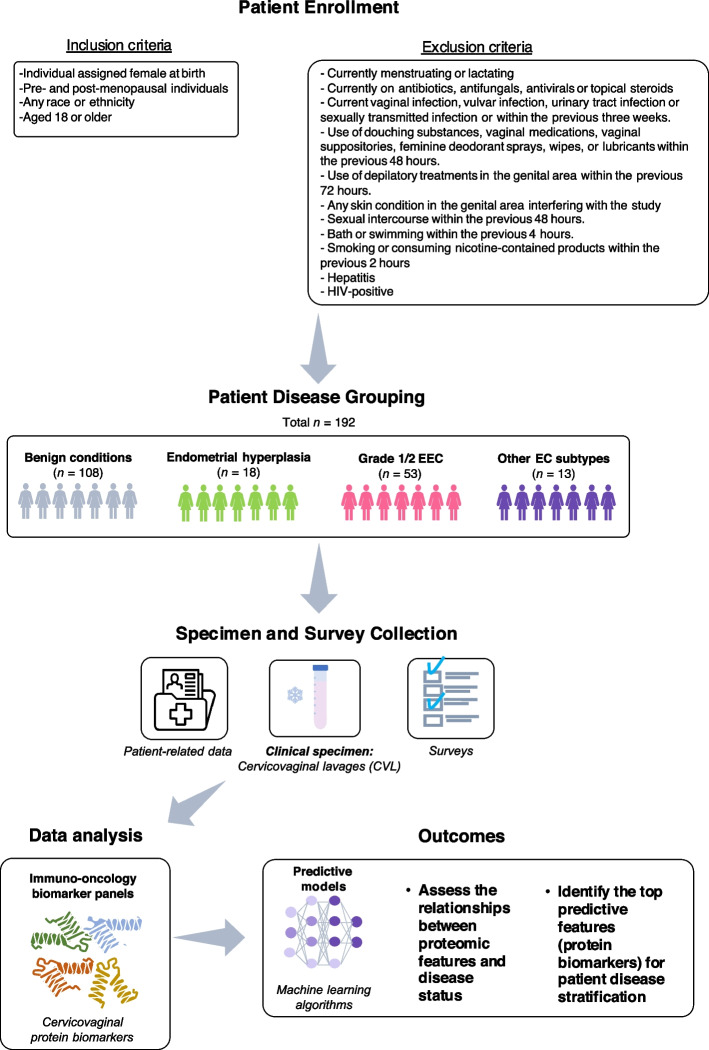
Study workflow. The study workflow is summarized here. The full inclusion and exclusion criteria are included. Briefly, we enrolled a total of 192 women. Based on histopathological results, 108 patients had benign conditions, 18 patients had endometrial hyperplasia and 66 had EC. The EC group was further stratified into grade 1 or 2 endometrial endometrioid carcinoma (EEC) (*n* = 53) and other EC (*n* = 13), which included grade 3 EEC and other non-endometrioid histopathological subtypes

Patient inclusion and exclusion criteria are outlined in Fig. 1. Patient characteristics and demographics are summarized in Table 1 and detailed patient characteristics and demographics can be found in Supplementary Table S1. This cohort was previously described by Łaniewski et al. [29]. Briefly, patients undergoing hysterectomy were stratified by disease groups: benign conditions (*n* = 108), endometrial hyperplasia (*n* = 18), grade 1/2 EEC (*n* = 53) and other EC types (*n* = 13) based on histopathology of tissue collected post hysterectomy. The average age of the cohort was 51 years old; the average BMI was 34.8 kg/m^2^. Most patients were Caucasian (74.7%), and our study included a relatively high percentage (26.2%) of patients identifying as Hispanic. Overall statistical (*p* < 0.0001) difference in the mean age of women was observed between disease groups. Specifically, patients with grade 1/2 EEC (58.73 years) and other EC types (60.77 years) were older than patients with benign conditions (45.55 years) (*p* < 0.0001). Endometrial hyperplasia patients (54.11 years) were also statistically (*p* < 0.01) older than patients with benign conditions. Furthermore, menopausal status significantly (*p* < 0.0001) differed between the disease groups. Of grade 1/2 EEC patients, 76.47% were postmenopausal, which was significantly (*p* < 0.0001) higher than the 43.16% of benign patients who were postmenopausal. When comparing the BMI of patients between the disease groups, endometrial hyperplasia patients (41.49 kg/m^2^) and grade 1/2 EEC patients (40.29 kg/m^2^) had significantly (*p* < 0.0001) higher BMI than benign patients (30.63 kg/m^2^). Additionally, we observed that endometrial hyperplasia patients had a statistically (*p* < 0.005) higher mean BMI than grade 1/2 EEC patients.

**Table 1:** Patient demographics and characteristics of the study cohort

Results	All	Benign conditions	Endometrial hyperplasia	Grade 1/2 EEC	Other EC types	*P* value
	(*n* = 192)	(*n* = 108)	(*n* = 18)	(*n* = 53)	(*n* = 13)	
**Age (mean (SD))** (*n* = 192)	51.02 (12.45)	45.55 (10.01)	54.11 (13.35)	58.73 (11.82)	60.77 (8.06)	< 0.0001
**Race** (***n* (%))** (*n* = 190)						0.004
White/Caucasian	142 (74.74)	78 (72.90)	16 (88.89)	37 (71.15)	11 (84.62)	
American Indian/Alaska Native	15 (7.89)	5 (4.67)	1 (5.56)	8 (15.38)	1 (7.69)	
Black/African American	12 (6.32)	11 (10.28)	0 (0.00)	1 (1.92)	0 (0.00)	
Mixed/Multi-racial	9 (4.74)	7 (6.54)	0 (0.00)	2 (3.95)	0 (0.00)	
Asian/Far East/South East	4 (2.11)	4 (3.74)	0 (0.00)	0 (0.00)	0 (0.00)	
Asian/Indian	1 (0.53)	0 (0.00)	1 (5.56)	0 (0.00)	0 (0.00)	
Native Hawaiian/Pacific Islander	1 (0.53)	0 (0.00)	0 (0.00)	1 (1.92)	0 (0.00)	
Middle Eastern/North African	1 (0.53)	0 (0.00)	0 (0.00)	0 (0.00)	1 (7.69)	
Not specified/other	5 (2.63)	2 (1.87)	0 (0.00)	3 (5.77)	0 (0.00)	
**Ethnicity (*n* (%))** (*n* = 191)						
Non-Hispanic	141 (73.82)	76 (70.37)	14 (77.78)	41 (78.85)	10 (76.92)	0.67
Hispanic	50 (26.18)	32 (29.63)	4 (22.22)	11 (21.15)	3 (23.08)	
**BMI (*n* (%))** (*n* = 192)						
< 25	29 (15.38)	23 (21.30)	0 (0.00)	4 (7.55)	2 (15.38)	< 0.0001
25–29	47 (24.38)	38 (35.19)	1 (5.56)	6 (11.32)	2 (15.38)	
30–34	30 (15.63)	19 (17.59)	2 (11.11)	6 (11.32)	3 (23.08)	
≥ 35	86 (44.79)	28 (25.93)	15 (83.33)	37 (69.81)	6 (46.15)	
**BMI (mean (SD))** (*n* = 192)	34.76(10.16)	30.63 (7.54)	41.49 (7.45)	40.29 (11.07)	37.22 (12.76)	< 0.0001
**Menopause status (*n* (%))** (*n* = 190)						
Premenopausal	108 (56.84)	89 (82.41)	6 (33.33)	12 (23.53)	1 (7.69)	< 0.0001
Postmenopausal	82 (43.16)	19 (17.59)	12 (66.67)	39 (76.47)	12 (92.31)	

Patient demographics and characteristics are summarized here. Kruskal-Wallis test was used to calculate *p* values for continuous variables and Fisher's exact test for categorical variables. Data on patients' ethnicity were available for 191, and race and menopausal status data were available for 190 patients. Taken from Łaniewski et al. [29]

### Global cervicovaginal protein profiles reveal distinct differences between endometrial cancer and benign patients

**Fig. 2:**
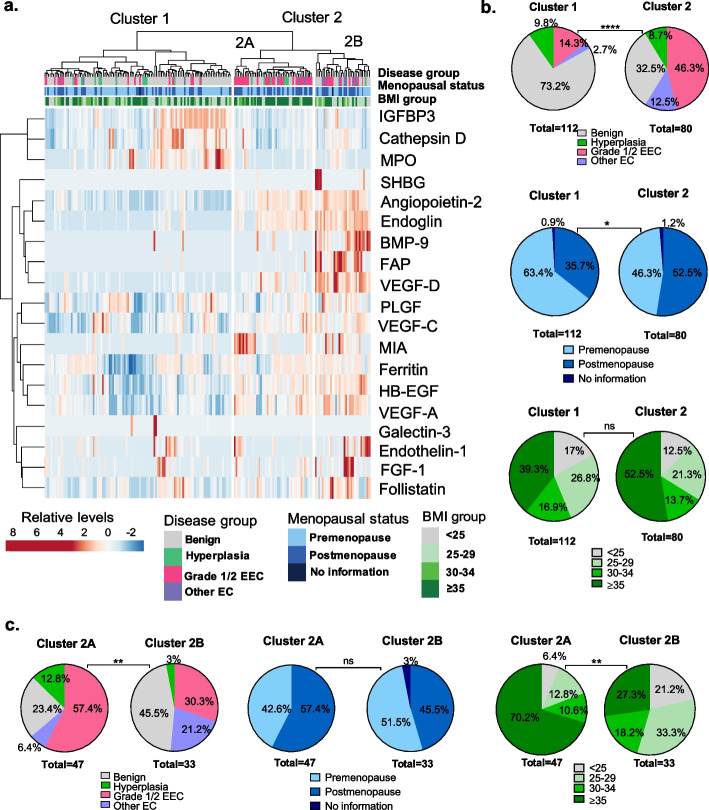
Unsupervised hierarchical clustering reveals two distinct patient clusters based on their cervicovaginal protein levels. **a** Heatmap depicts the relative levels of proteins across all samples. Prior to analysis, data were mean-centered, and variance scaled along each row. Euclidean distance and Ward linkage method were used for clustering. The analysis revealed two distinct patient clusters. Pie charts illustrate the patient demographics of the two clusters **b**, based on disease group, menopausal status**,** and BMI group. Disease group and menopausal status were statistically different among the clusters. Subcluster analysis of cluster 2 illustrated disease and BMI group were statistically different between cluster 2A and 2B **c**. Statistical difference was assessed using the chi-square test. Asterisks indicate the *p* value (* *p* < 0.05, **** *p* < 0.0001, ns (not significant))

To visualize the cervicovaginal protein profiles of patients, an unsupervised hierarchical clustering analysis was conducted, and a heatmap with a dendrogram (Fig. 2a) displayed two separate patient clusters based on global protein levels. Overall, cluster 1 consisted of relatively low levels of the proteins, and cluster 2 contained relatively high levels of the proteins. To better understand the characteristics of patients belonging to these two distinct clusters, we overlayed patient-related data such as disease group, BMI group, and menopausal status and analyzed the statistical differences in these characteristics between the two clusters. Disease group (Fig. 2b) and menopausal status significantly differed between the two clusters (*p* < 0.0001 and *p* < 0.05, respectively). However, the BMI groups did not statistically (*p* = 0.34) vary between the two clusters. Briefly, cluster 1 predominantly consisted of benign (73.2%) and premenopausal (63.4%) patients. In contrast, cluster 2, consisted of mainly patients with EC (58.8%), and 52.5% of cluster 2 were postmenopausal patients. Further analysis of cluster 2 revealed two subclusters, 2A and 2B (Fig. 2c). Regarding relative protein levels, subcluster 2B exhibited higher levels of cervicovaginal proteins compared to subcluster 2A. When analyzing patient characteristics, subcluster 2A contained significantly (*p* = 0.0097) more grade 1/2 EEC (57.4%) than subcluster 2B (30.3%), and subcluster 2B contained significantly more other EC subtypes (21.2%) than subcluster 2A (6.4%). Additionally, subcluster 2A included significantly (*p* = 0.0013) more patients with a BMI of equal to or above 35 (70.2%) than subcluster 2B (27.3%). Overall, unsupervised hierarchical clustering revealed EC patients had altered global protein levels compared to benign patients.

### Cervicovaginal growth and angiogenic factor levels are increased with EC severity

**Fig. 3:**
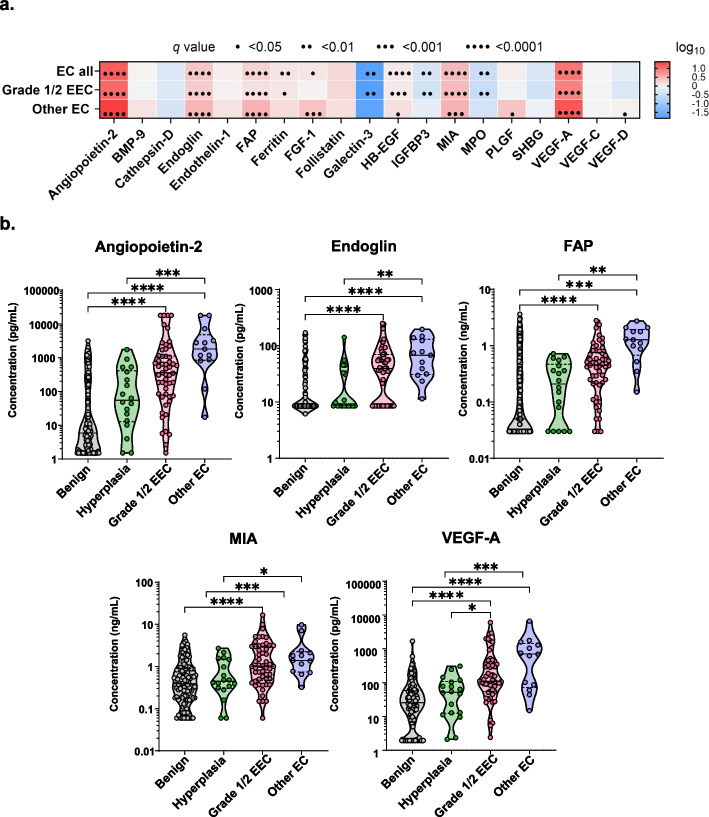
Numerous growth and angiogenic markers are significantly upregulated in patients with EC**. a** A heatmap demonstrates proteins that are statistically up- or downregulated (fold change (FC)) in endometrial cancer (*n* = 66), grade 1/2 EEC (*n* = 53), or other EC types (*n* = 13) compared to benign patients. Statistical difference was assessed using a two-sample *t*-test with FDR correction. Asterisks indicate the *q* value (* *q* < 0.05, ** *q* < 0.01, *** *q* < 0.001, **** *q* < 0.0001). **b** Truncated violin plots depict the concentration of key growth factors are statistically elevated in grade 1/2 EEC and other EC types compared to benign patients. *P* values were calculated using a 1-way ANOVA with Bonferroni’s correction. The horizontal line represents the median value, and the asterisks indicate the *p* value (* *p* < 0.05, ** *p* < 0.01, *** *p* < 0.001, **** *p* < 0.0001)

To detect differences in protein levels between EC and benign patients, a fold change (FC) was calculated, and statistical differences were assessed. We compared EC all, grade 1/2 EEC, and other EC to benign patients (Fig. 3a) to identify individual proteins that significantly differed between these disease groups. When comparing EC all to benign patients, we identified 11 out of 19 proteins to be significantly different after FDR correction at 5%. Of these, eight proteins exhibited significant upregulation in EC all: angiopoietin-2 (*q* < 0.0001), endoglin (*q* < 0.0001), FAP (*q* < 0.0001), ferritin (*q* = 0.004), FGF-1 (*q* = 0.02), MIA (*q* < 0.0001), HB-EGF (*q* < 0.0001), and VEGF-A (*q* < 0.0001). In contrast, three proteins were significantly (*q* < 0.01) downregulated in EC all, including galectin-3, MPO, and IGFBP3. When analyzing the differences between EC subtypes and benign patients, all previously mentioned proteins for EC all, except FGF-1, were also significantly altered in grade 1/2 EEC compared to benign patients.

We identified nine proteins to be significantly (*q* ranging from 0.02 to < 0.0001) upregulated in other EC types, including angiopoietin-2, endoglin, FAP, FGF-1, HB-EGF, MIA, PLGF, VEGF-A, and VEGF-D. Notably, angiopoietin-2, endoglin, FAP, and VEGF-A were significantly (*q* < 0.0001) upregulated in all EC groups: EC all, grade 1/2 EEC, and other EC types. Furthermore, angiopoietin-2, endoglin, FAP, MIA, and VEGF-A exhibited higher FC in other EC types compared to grade 1/2 EEC (Fig. 3A). In the analysis of the protein levels across all the disease groups (Fig. 3b and Fig. S1), angiopoietin-2, endoglin, FAP, MIA, and VEGF-A levels were significantly (*p* < 0.05- < 0.001) higher in other EC patients compared to endometrial hyperplasia patients. Of those, only VEGF-A levels were statistically (*p* < 0.05) elevated in grade 1/2 EEC compared to endometrial hyperplasia patients. Although not statistically significant, a noticeable trend was observed, in which the levels of angiopoietin-2, endoglin, FAP, MIA, and VEGF-A were increased with the severity of disease. These results highlighted some key proteins in CVLs that could be utilized to differentiate EC patients from benign patients.

### Growth and angiogenic markers exhibit biomarker potential in endometrial cancer

**Fig. 4:**
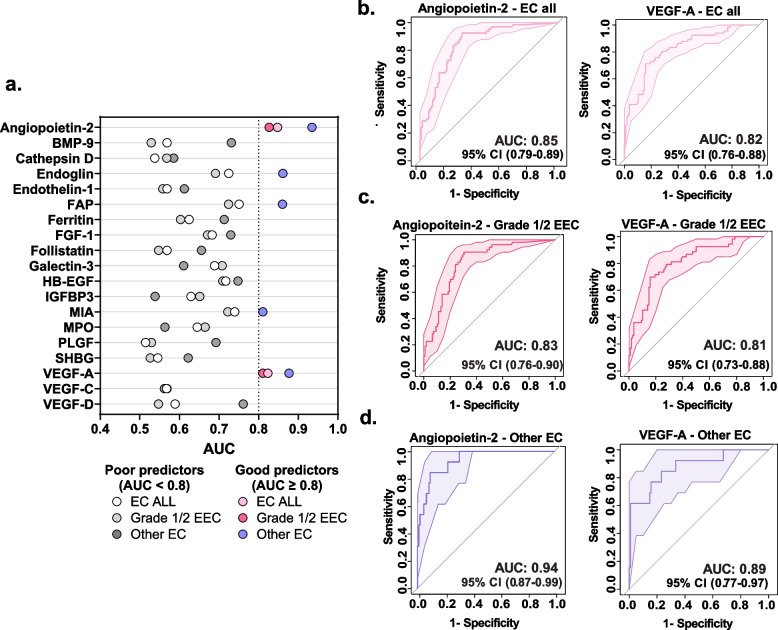
Growth factors in cervicovaginal lavage show good to excellent discriminatory potential for endometrial cancer**. a** Univariate ROC analysis assessed the potential of proteins for discriminating EC all, grade 1/2 EEC, and other EC types from benign patients. Angiopoietin-2 and VEGF-A have good discriminatory potential for all endometrial cancers **b**, grade 1/2 EEC **c** and angiopoietin-2 has excellent discriminatory potential for other EC types **d**. In addition, endoglin, FAP, MIA, and VEGF-A have good discriminatory potential for other EC types. The discriminatory potential was based on the area under the curve (AUC) values. Proteins with an AUC of above or equal to 0.8 were considered good discriminators, and proteins with an AUC of above or equal to 0.9 were considered excellent discriminators

Next, we utilized univariate receiver operating characteristic (ROC) analysis to identify proteins exhibiting high sensitivity and specificity for EC all, grade 1/2 EEC, and other EC types compared to benign conditions. A ROC curve was plotted based on the sensitivity and specificity values of the proteins, and the area under the curve (AUC) was used to measure the strength of the discriminators. A protein with an AUC above or equal to 0.8 is considered a good discriminator, and a protein with an AUC above 0.9 is regarded as an excellent discriminator. Firstly, we compared EC all patients to benign patients and identified two proteins as good discriminators for EC all: angiopoietin-2 and VEGF-A (Fig. 4a), with an AUC of 0.85 and 0.82, respectively (Fig. 4b). When comparing grade 1/2 EEC patients to benign patients, angiopoietin-2 and VEGF-A also exhibited a good discriminatory potential (Fig. 4c), with an AUC of 0.83 and 0.81, respectively. Finally, when examining the ROC for other EC types versus benign patients, we identified four proteins with a good discriminatory potential (Fig. 4d and Fig. S2): endoglin (AUC of 0.86), FAP (AUC of 0.86), MIA (AUC of 0.81), VEGF-A (AUC of 0.89), and one protein with an excellent discriminatory potential: angiopoietin-2 (AUC of 0.94) (Fig. 4d). Overall, univariate biomarker discovery analysis revealed several individual proteins with diagnostic potential for EC.

### Multivariate protein model for EC diagnosis illustrates excellent discriminatory potential

**Fig. 5:**
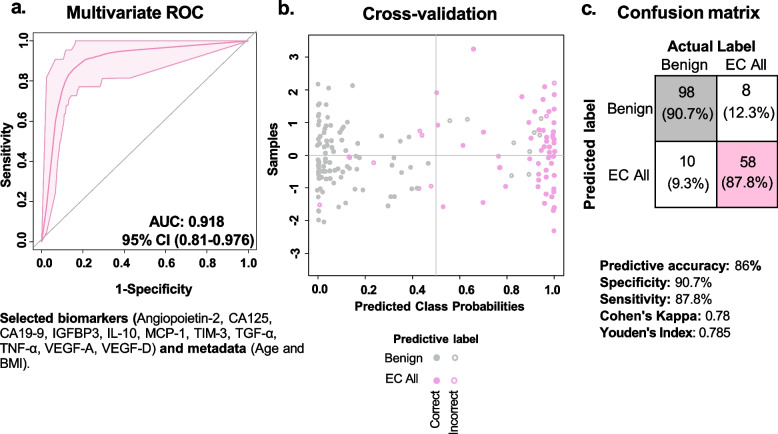
ROC analysis demonstrates the diagnostic utility of a multivariate protein model with metadata for EC.** a** Multivariate ROC curve analysis resulted in an excellent AUC of 0.918. To build the model, 11 proteins were selected based on high least absolute shrinkage and selection operator (LASSO) frequencies and empirical testing. Age and BMI were added to the model as they are known risk factors for EC. CA125, CA19-9, IL-10, MCP-1, TIM-3, TGF-α, TNF-α, and VEGF-A data were previously evaluated in Łaniewski et al. [29]. **b** A scatter plot illustrates the Monte Carlo cross-validation (MCCV) with a predictive accuracy of 86%. The predicted class probabilities of the samples are shown, using the classifier at a threshold of 0.5. **c** The confusion matrix depicts the number of times this combination of proteins accurately classified patients to the disease groups. This multivariate model accurately classified 156/174 samples with a specificity of 90.7% and a sensitivity of 87.8%

Next, we utilized a multivariate approach and assessed combinations of proteins to increase correct EC classification. Using multivariate ROC analyses, we compared EC all patients to benign patients using a logistic regression model (Fig. 5a). Proteins with high least absolute shrinkage and selection operator (LASSO) frequencies were selected and the model was refined through empirical testing of several protein combinations. Age and BMI were added to the model as they are known risk factors for EC. We identified a combination of angiopoietin-2, IGFBP3 and VEGF-D, with previously evaluated biomarkers [29]: carbohydrate antigen (CA)-125, carbohydrate antigen (CA)-19–9, interleukin (IL)-10, monocyte chemoattractant protein (MCP)-1, T cell immunoglobulin and mucin-domain containing (TIM)-3, transforming growth factor (TGF)-α, tumor necrosis factor (TNF)-α, VEGF-A. This biomarker and metadata combination generated an AUC of 0.918, which is indicative of an excellent discriminatory potential. The Monte Carlo cross-validation was utilized to assess the predictive accuracy, and a confusion matrix showing correct and incorrect disease classification was generated. This combination of proteins demonstrated a predictive accuracy of 86% (Fig. 5b), with a specificity of 90.7% and a sensitivity of 87.8% (Fig. 4c). Overall, this multivariate protein model accurately classified 156 of 174 patients tested, including 98 of the 108 benign patients and 58 of the 66 EC patients. Cohen’s kappa was calculated to be 0.78 which shows a substantial agreement between the actual label and the predictive label, and Youden’s index was calculated to be 0.785. After removing age and BMI from the protein model (Fig. S3), we found the AUC of the model increased to 0.923; however, the predictive accuracy decreased to 83.7%. Furthermore, the specificity and sensitivity decreased to 87% and 86.4%, respectively. These analyses revealed that the multivariate protein model with patient metadata can accurately discriminate EC from benign conditions.

### Cervicovaginal growth and angiogenic markers demonstrate prognostic utility in endometrial cancer

**Fig. 6:**
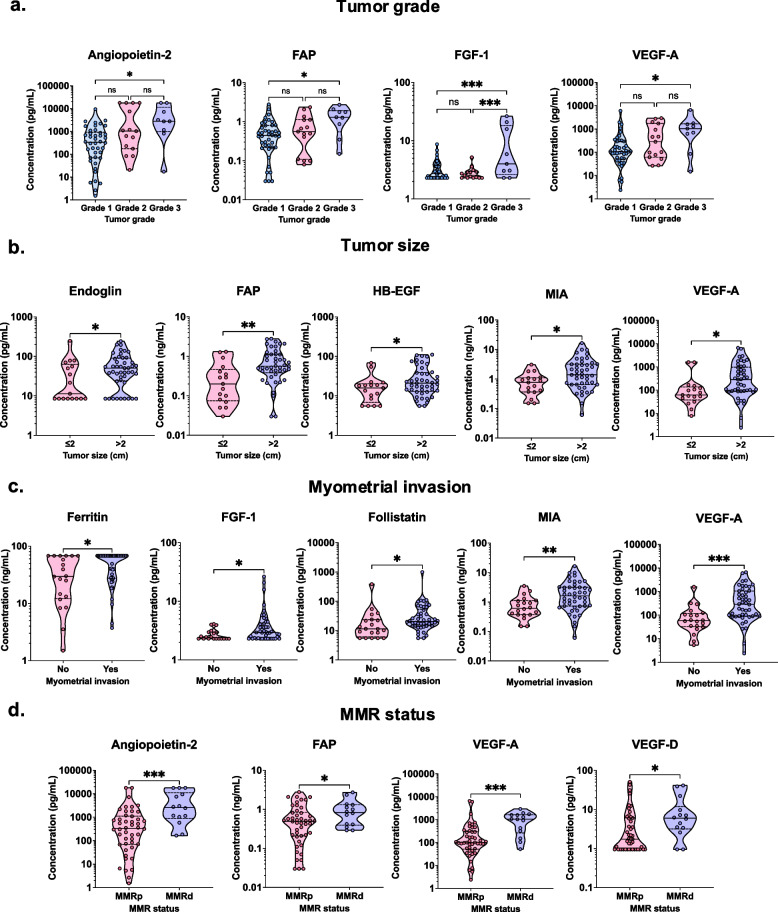
Key growth factors in cervicovaginal lavages align with tumor characteristics demonstrating prognostic utility**.** Truncated violin plots depict several proteins are elevated in patients with more advanced tumor grade **a**, larger tumor size **b**, in tumors with a presence of myometrial invasion **c**, and MMR deficiency **d**. *P* values were calculated using Student’s *t*-test or a 1-way ANOVA with Bonferroni’s correction and asterisks indicate the *p* value (* *p* < 0.05, ** *p* < 0.01, *** *p* < 0.001, **** *p* < 0.0001)

Lastly, to assess the prognostic utility of the proteins measured in CVLs, we evaluated the relationship between protein levels and tumor characteristics, such as tumor grade, tumor size, myometrial invasion, and mismatch repair (MMR) status. Data were also collected on the FIGO stage and the presence of lymphovascular invasion; however, we were unable to analyze these patient factors due to the unbalanced distribution, with the majority of tumors being assigned FIGO stage I (87.1%) and having an absence of lymphovascular invasion (93.8%) (Table S2). When EC patients were classified based on tumor grade (Fig. 6a), significant elevation in the levels of angiopoietin-2 (*p* = 0.015), FAP (*p* = 0.033), FGF-1 (*p* = 0.0004), and VEGF-A (*p* = 0.018) were observed in the more advanced grade 3 tumors compared to grade 1 tumors. Interestingly, although not significant, we observed a gradual increase in angiopoietin-2, FAP, and VEGF-A levels, as tumor grade increased. When stratifying EC patients based on tumor size (Fig. 6b), five proteins were significantly increased in patients with tumors larger than 2 cm: endoglin (*p* = 0.03), FAP (*p* = 0.001), HB-EGF (*p* = 0.04), MIA (*p* = 0.01), and VEGF-A (*p* = 0.02), when compared to patients with smaller tumors (≤ 2 cm). Furthermore, through correlation analysis, we demonstrated that concentrations of BMP-9, FAP, follistatin, MIA, and VEGF-A significantly (*p* ranging from 0.041 to 0.0012) correlate with tumor size (measured in cm) (Fig. S4). We also assessed the protein levels between tumors with or without myometrial invasion (Fig. 6c). We observed the levels of five proteins: ferritin (*p* = 0.03), FGF-1 (*p* = 0.03), follistatin (*p* = 0.04), MIA (*p* = 0.0043), and VEGF-A (*p* = 0.0009), were significantly higher in patients with tumors that invaded the myometrium compared to those with tumors that did not. When assessing the correlation of protein levels and myometrial invasion (measured in mm), six proteins: cathepsin-D, endothelin-1, FGF-1, follistatin, MIA, PLGF, and VEGF-A significantly (*p* ranging from 0.04 to < 0.0001) correlated with the depth of myometrial invasion (Fig. S4). Only follistatin and MIA significantly (*p* ranging from 0.04 to < 0.0001) correlated with tumor size and myometrial invasion depth. Patients with MMR deficient (MMRd) tumors exhibited significantly higher levels of angiopoietin-2 (*p* = 0.0008), FAP (*p* = 0.04), VEGF-A (*p* = 0.0004), and VEGF-D (*p* = 0.02), than patients with MMR proficient (MMRp) tumors (Fig. 6d). In summary, VEGF-A aligned with all the tumor characteristics we evaluated, FAP was associated with tumor grade, size, and MMR status, and angiopoietin-2 was associated with tumor grade and MMR status. The growth and angiogenic markers were then correlated with a further 72 pro- and anti-inflammatory cytokines, chemokines, hormones, immune checkpoints and apoptosis-related proteins previously reported on in this cohort [29] (Fig. S5). Analysis revealed strong positive correlations (correlation coefficient > 0.7) among growth factors, pro- and anti-inflammatory cytokines, chemokines, hormones and immune checkpoint proteins, including proteins identified in the multivariate protein model such as angiopoietin-2, IL-10, TNF-α and TIM-3. Furthermore, when analyzing the correlation of angiopoietin-2, FAP, and VEGF concentrations in EC and benign patients (Fig. S6a and b), we identified that these proteins significantly correlated with one another. Our analysis illustrates the utility of proteins measured in lower reproductive tract samples as diagnostic and prognostic markers for the upper reproductive tract disease, EC.

## Discussion

As the rates of EC are continually increasing, including in younger females, there is a pressing need for early detection tools to facilitate timely treatment and improved disease outcomes, thus allowing for the possibility of fertility-sparing options in those being diagnosed at a younger age. Current diagnostic approaches for EC are consistently reported to cause anxiety and pain [18], and diagnosis requires numerous procedures. Consequently, these could serve as barriers to EC detection, particularly in rural-based populations and those with limited access to healthcare [2, 31–33]. A recent study examining healthcare utilization of females diagnosed with EC found those earning less than $25,000, unsurprisingly, had more barriers to access to care; these included anxiety towards seeing a provider, paying out of pocket, and transportation issues [33].

In this study, we expanded on our previous study [29], in which we demonstrated CVLs as a promising collection technique for EC biomarker detection. Herein, we focused on a panel of multiple growth factors and angiogenic markers measured in CVL samples and exhibited the ability to increase the diagnostic and prognostic utility of CVLs. Further, we identified several individual proteins and a novel multivariate protein model for EC detection. Unlike traditional diagnostic methods for EC, CVLs offer a minimally invasive approach due to the anatomical continuity of the female reproductive tract and hold promise for future EC diagnostics [28]. Our novel multivariate protein biomarker model, in combination with innovative CVL sampling, may enable earlier diagnosis, as CVLs could be utilized in routine gynecological visits, thus facilitating earlier detection and intervention. This is particularly important considering rates of EC are increasing in younger females [5], hence, early detection and diagnosis could allow for fertility-sparing treatment options. While other detection methods such as serum- and tissue-based biomarkers have been assessed, their potential is limited by low sensitivity and specificity for EC. For example, serum HE4 has been studied in the context of EC, yet a meta-analysis by Li et al. [34] found that the total pooled sensitivity and specificity, and AUC of 23 studies were 65%, 91%, and 0.84, respectively. Another blood-based biomarker that has been assessed for EC detection is CA125. A meta-analysis by Li et al. [27] demonstrated that CA125 had a pooled sensitivity of 35%, a specificity of 83%, and an AUC of 0.58 in diagnosing EC. This suggests that blood-based biomarkers might be limited in their capabilities for EC, possibly due to the relatively low concentrations of these markers in peripheral blood samples [28]. Currently, an endometrial biopsy with or without hysteroscopy or dilation and curettage is required to confirm the presence of EC. To determine whether an endometrial biopsy is needed, physicians mostly utilize a TVUS to measure endometrial thickness. While TVUS procedures have high patient acceptability and are well-tolerated [35–37], the TVUS lacks high sensitivity for EC. In a simulated cohort, the TVUS exhibited only fair discriminatory properties for EC detection (AUC of 0.73) in White females but poor discriminatory properties (AUC of 0.57) for Black females [22], demonstrating racial disparities in the ability of this tool to diagnose EC. Comparatively, our multivariate protein model of 11 proteins and metadata (age and BMI) for EC had an overall excellent discriminatory potential (AUC of 0.918). Assessment of the TVUS diagnostic sensitivity and specificity in EC varies due to differing opinions on optimal endometrial thickness for diagnosis, and differences in operator use. TVUS sensitivity and specificity values vary from 54.1–80.5% and 80.7–97.2% [38–40], respectively. In comparison, our multivariate protein biomarker model has a sensitivity of 87.7% and a specificity of 90.7%. Interestingly, we found that adding patient-related metadata such as age and BMI increased the overall predictive accuracy, specificity, and sensitivity of the model, compared to the multivariate model with the 11 proteins only.

Throughout our analyses, angiopoietin-2 has displayed robust diagnostic capabilities for all the EC types we analyzed, exhibiting the largest concentration differences, as well as highest specificity and sensitivity for EC of all the proteins we tested in CVLs. Angiopoietin-2 plays a crucial role in angiogenesis. Previous research has shown that angiopoietin-2 is secreted by endothelial cells, via Weibel-Palade bodies [41] or exosomes [42]. Notably, Xie et al. [43] demonstrated in hepatocellular carcinoma, that angiopoietin-2 can be secreted by endothelial cells via tumor-derived exosomes, though, this pathway has not been investigated in the context of EC. The ratio of angiopoietin-1/angiopoietin-2 at the Tie-2 vascular endothelial receptor [44] promotes mature vessel growth and retains tight endothelial cell interactions [45]. However, under the hypoxic conditions caused by tumors, endothelial cells overexpress angiopoietin-2 [46]. This coupled with increased expression of VEGF-A, stimulated by hypoxic inducible factor (HIF)-1α [47] causes pro-angiogenic effects in EC, related to tumor growth and angiogenesis. This could explain the differences observed with higher levels of angiopoietin-2 in other EC types compared to the grade 1/2 EEC patients. In fact, research in breast cancer has demonstrated that angiopoietin-2 can stimulate tumor migration and invasion through Tie-2 independent signaling [48]. Imanishi et al. exhibited that angiopoietin-2 activates α_5_β_1_, which results in the activation of ILK/Akt, and GSK 3β/Snail/E-cadherin and thus promotes tumor cell motility [48]. Though further research in EC is required to elucidate whether angiopoetin-2 can act in a Tie-2-independent manner. Previously, Holland et al. [49] displayed an increased abundance of angiopoietin-2 mRNA transcripts in EC tissue samples when compared to the benign postmenopausal endometrium. High serum levels of angiopoietin-2 have previously been associated with severity of hepatocellular carcinoma [50], colorectal cancer [51] and metastatic melanoma [52]. Angiopoietin-2 also exhibited prognostic utility, as we observed a significant increase in the cervicovaginal levels of angiopoietin-2 in patients with MMRd tumors compared to MMRp tumors. In contrast to our findings, Jary et al. [53] displayed that angiopoietin-2 did not relate to microsatellite instability (also known as MMRd) in colorectal tumors, which suggests this relationship could be cancer-specific. Thus, further research into the role of angiopoietin-2 in the context of DNA repair mechanisms is warranted. Additionally, we observed a trend in the levels of angiopoietin-2 and tumor grade; as tumor grade increased, the levels of angiopoietin-2 increased, indicating an association with tumor progression and aggressiveness. Previously, angiopoietin-2 has been linked to more severe FIGO stages [54]; however, in this study, we were unable to analyze the FIGO stage due to the unbalanced distribution of the stages in our cohort. Terlikowska et al. [54] also analyzed angiopoietin-2 levels in the context of tumor grade in EC and did not observe the trend that we demonstrated. Although it is important to note they measured angiopoietin-2 in serum samples and did not have any grade 3 tumors in their study.

Another key protein that we identified in our analyses was FAP. FAP plays a vital role in tissue remodeling and has demonstrated an ability to support tumor cell development [55]. FAP overexpression has been noted in multiple malignancies, including breast cancer [56], gastric cancer [57], and colorectal cancer [58]. FAP is expressed on cancer-associated fibroblasts (CAFs) [59]. CAFs have exhibited pro-tumorigenic effects in EC [60], including increasing the secretion of VEGF-A to induce EC cell proliferation [61]. Notably, in vitro*,* xenograft model indicated that CAFs can facilitate tumor progression through the IL-10/JAK1/STAT3 signaling pathway [61]. This could explain the observed differences in concentrations of FAP between grade 1/2 EEC patients and other EC type patients. Interestingly, Łaniewski et al. [29] demonstrated an increased concentration of IL-10 within the EC group of this cohort. In this study, we found FAP to be significantly increased in both grade 1/2 EEC and other EC types compared to the benign group. However, univariate ROC analysis revealed that FAP only had fair discriminatory potential for grade 1/2 EEC, in comparison to other EC types in which FAP displayed good discriminatory potential, suggesting FAP may be a marker for more advanced EC. Furthermore, when analyzing the prognostic ability of FAP, we found FAP to be significantly elevated in grade 3 tumors, larger tumors and MMRd tumors further suggesting FAP’s role in more advanced EC. Yet, research assessing FAP as a prognostic marker for EC is limited. One study demonstrated that CAFs highly expressing FAP correlated with EC invasion and migration through in vitro analysis [60]; although, we did not observe the same association of FAP levels with myometrial invasion in our clinical data. Another study illustrated that a subpopulation of CAFs was associated with an unfavorable prognosis and a low response to therapy in bladder cancer [62]. The role of CAF subpopulations in EC requires further investigation.

Another growth factor that demonstrated strong diagnostic and prognostic value in EC is VEGF-A. Of all the proteins we analyzed, VEGF-A was the only protein to significantly align with all unfavorable tumor characteristics we assessed (tumor size, tumor grade, myometrial invasion, and MMR status). Tumor cells secrete VEGF-A to promote survival and proliferation through angiogenesis [63]. Numerous studies have demonstrated VEGF-A to be significantly upregulated in EC [49, 64–67]. In the context of tumor characteristics, similarly to our report, few studies [68–72] displayed serum levels of VEGF-A correlated with tumor grade. Furthermore, Abbink et al. [71] demonstrated that VEGF-A serum levels were elevated in patients with myometrial invasion, in line with our results. Herein, we also exhibited VEGF-A to be significantly higher in MMRd EC tumors compared to MMRp tumors. Interestingly, some studies on colorectal cancer also found VEGF-A levels to be significantly higher in MMRd tumors compared to MMRp [73, 74]. Although, another study on colorectal cancer showed conflicting results [75]. Nevertheless, VEGF-A could prove useful in the context of MMR status in EC; however, further research is required to validate this finding. In addition, VEGF-A concentrations trended higher in other EC patients than in grade 1/2 EEC, however, this difference did not reach significance. As our data on the tumor characteristics suggests, VEGF-A holds strong prognostic value. Mechanistically, as growing tumors generate hypoxic conditions, which in turn stimulates HIF-1α [47], HIF-1α directly increases VEGF-A expression by binding to the VEGF-A gene promoter [76]. VEGF-A binds to receptor tyrosine kinase, VEGFR-2. Phosphorylation of VEGFR-2 leads to the activation of numerous downstream pathways, including the phosphoinositide 3-kinase (PI3K) pathway. The PI3K pathway has been shown to promote endothelial cell survival, proliferation and angiogenesis [77]. Thus, this could explain the differences we observed in the EC types in our cohort.

Interestingly, our clinical data suggests a possible synergistic pathophysiological mechanism between a few of the key proteins (angiopoietin-2, FAP, and VEGF-A). We illustrated that, in EC patients, cervicovaginal levels of VEGF-A and FAP strongly correlate with angiopoietin-2. It is well known that angiopoietin-2 and VEGF-A act synergistically to promote angiogenesis in EC. High levels of angiopoietin-2 in combination with VEGF-A under the hypoxic conditions caused by tumors result in vessel destabilization and immature vessel sprouting [78]. Saito et al. [79] found high levels of angiopoietin-2 in combination with high VEGF-A levels increased the total vessel count and the microvessel count in EC. Furthermore, the same study revealed that the vessel density was smaller in samples with high angiopoietin-2 and VEGF-A compared to those with high angiopoietin-1 [79]. An in vitro analysis in an ovarian cancer cell line found that angiopoietin-2 can stimulate the accumulation of CAFs [80]. Our clinical data suggests a similar mechanism in EC. However, further research is needed to confirm this finding. Subramaniam et al. [61] also demonstrated that CAFs increase the secretion of VEGF-A in EC and have a pro-angiogenic role in EC. Intriguingly, in our study, we also observed a positive correlation between FAP and angiopoietin-2 in benign patients. This suggests FAP and angiopoietin-2 may play a role in the pathophysiology of one of the benign gynecological conditions. FAP has been found to be elevated in fibroids [81], though, there is limited literature assessing angiopoietin-2 in fibroids, one study found no differences in angiopoietin-2 gene allele frequency between uterine fibroids patients and controls [82]. It is important to note the overall concentrations are higher in the EC group compared to the benign group.

Strengths of our study include a relatively large cohort size of 192 participants, which included 66 EC patients. Additionally, all samples were collected at the time of hysterectomy for consistency across the cohort. Therefore, our coparison group included patients with benign gynecologic conditions such as adenomyosis, endometriosis, and fibroids. These gynecologic conditions are prevalent in the general population: the estimated occurrence of adenomyosis in symptomatic patients ranges from 30–35% [83]; endometriosis affects around 10% of reproductive-aged females [84], and up to 80% of Black females and up to 70% of White females are diagnosed with fibroids during their reproductive years [85]. Hence, we consider this to be a strength in our study as we were still able to discriminate EC from benign gynecologic conditions with a high predictive accuracy. However, our study has few limitations. Further biomarker validation is needed in a larger cohort of patients, as well as in patients not undergoing hysterectomy. Our cohort did contain a small number of individuals diagnosed with endometrial hyperplasia, which limited our ability to conduct protein biomarker discovery for this precancerous condition, thus future studies should also include a larger number of endometrial hyperplasia patients. Due to the unbalanced distribution of FIGO stage in our cohort, with 87.1% of EC patients assigned stage I tumors, we were unable to assess protein levels related to the stage of EC. We also acknowledge the limited number of patients with more advanced stage EC; nevertheless, we were still able to demonstrate the diagnostic potential of these proteins for different EC subtypes. Thus, utilizing these proteins to discriminate early-stage tumors from the benign group is a strength of our study. Future aims include validation of our multivariate protein model in larger cohorts.

## Conclusion

In summary, our study illustrated the effectiveness of CVLs as a tool for EC detection. We specifically identified angiopoietin-2, endoglin, FAP, MIA and VEGF-A as biomarkers with significant diagnostic and prognostic potential for EC. Through multivariate ROC analysis, we found that an innovative biomarker model consisting of 11 growth and angiogenic markers in combination with patients’ age and BMI displayed excellent discriminatory potential for EC, showcasing the diagnostic utility of CVL sampling. Specifically, the multivariate protein model accurately classified patients with a sensitivity of 87.8% and a specificity of 90.7%, a Cohen’s Kappa of 0.78 and a Youden’s index of 0.785. Moreover, these growth factors, detected in the CVLs, related to tumor characteristics, such as tumor grade, size, myometrial invasion, and MMR status, suggesting their prognostic potential. Overall, utilizing minimally invasive and less time-consuming sampling techniques such as CVLs could improve equity in access to early diagnosis, thereby enhancing patient outcomes and reducing health disparities in EC.

## Materials and methods

### Patient enrollment

A total of 192 cis-gender women undergoing a hysterectomy for benign or malignant conditions were enrolled at three clinical sites across Phoenix, Arizona, USA: Banner University Medical Center-Phoenix, Dignity Health Chandler Regional Medical Center and Valleywise Health Medical Center. The patients were recruited from June 2018 to February 2020. Based on the histopathological results of hysterectomy samples, patients were categorized into one of the following groups: benign conditions (*n* = 108), endometrial hyperplasia (*n* = 18), or EC (*n* = 66). The EC group was further stratified into grade 1 or 2 endometrial endometrioid carcinoma (EEC) (*n* = 53) and other EC (*n* = 13), which included grade 3 EEC and other non-endometrioid histopathological subtypes. The inclusion and exclusion criteria were previously described in by Łaniewski et al. [29]. The exclusion criteria were confirmed through physician’s pelvic exam and medical records and/or self-reported.

### Sample collection

Prior to a hysterectomy procedure, CVL samples were collected by a surgeon, before vaginal sterilization, and after anesthesia. CVL samples were obtained with a non-lubricated speculum, and 10 mL of sterile 0.9% saline solution (Teknova, Hollister, CA). Samples were placed on ice and frozen at -80 °C within an hour of collection. Before downstream analyses, samples were processed as follows; samples were thawed on ice, centrifuged (700 × *g* at 4 °C for 10 min), aliquoted to limit multiple freeze–thaw cycles, and stored at -80 °C, as previously described [29].

### Soluble protein quantification

A total of 19 proteins were quantified in CVL samples using the Milliplex MAP Magnetic Bead Immunoassays: Human Circulating Cancer Biomarker Panel 3 and Human Angiogenesis/Growth Factor Panel 1 (Millipore, Billerica, MA) targeting the following proteins: angiopoietin-2, bone morphogenetic protein (BMP)-9, cathepsin-D, endoglin, endothelin-1, ferritin, fibroblast activation protein (FAP), fibroblast growth factor (FGF)-1, follistatin, galectin-3, heparin-binding epidermal growth factor (HB-EGF), insulin-like growth factor-binding protein (IGFBP)-3, melanoma inhibitory activity (MIA), myeloperoxidase (MPO), placental growth factor (PLGF), sex hormone binding globulin (SHBG), vascular endothelial growth factor (VEGF)-A, VEGF-C and VEGF-D. The Bio-Plex 200 instrument was used to collect data, and the Manager 5.0 software was utilized to analyze the data (Bio-Rad, Hercules, CA). To determine the protein concentrations, a five-parametric logistic regression curve fit was used. Each sample was analyzed in duplicate. Any concentration values below the minimum detectable concentration were replaced with half of the minimum detection limit as indicated in the manufacturer’s guidelines. Prior to data analysis, protein concentrations were log_10_-transformed before data analysis.

### Hierarchical clustering analysis

Unsupervised hierarchical clustering analysis was conducted to visualize the relationships between relative levels of protein biomarkers and patient characteristics such as body mass index (BMI), disease group, and menopausal status. Prior to analysis, protein concentrations were mean-centered and variance-scaled. The Euclidean distance measure and the Ward linkage method were applied for sample clustering. ClustVis web tool [86] was utilized for hierarchical clustering analysis.

### Fold change analysis

A fold change analysis was used to compare the absolute value of change of the means of each protein between the two disease groups. Multiple *t*-tests with false discovery rate (FDR) correction (*q* < 0.05) were utilized to determine significant differences in protein biomarker levels between disease groups. A heatmap was then used to visualize the data from fold change and two-sample *t*-test analyses. Proteins were significantly upregulated or downregulated based on a fold change (FC ≥ 2 or FC ≤ -2) and *q* value (*q* < 0.05).

### Univariate and multivariate receiver operating characteristics (ROC) analyses

A univariate ROC analysis was performed using Prism 9.0 (GraphPad Software, Boston, MA, USA) on each protein to identify biomarkers discriminating disease groups with high sensitivity and specificity. A ROC curve was generated, and the area under the curve (AUC) was calculated. Any proteins with an AUC of greater than or equal to 0.8 were considered good discriminatory biomarkers. Multivariate ROC was conducted using MetaboAnalyst 6.0 [87]. Protein biomarkers were selected based on high least absolute shrinkage and selection operator (LASSO) frequencies and empirical testing of combinations of proteins. A Monte Carlo cross-validation model was utilized to assess the performance of the multivariate model, using 2/3 of the samples for training and 1/3 of the samples for testing. A ROC curve was generated from the averaged results of 100 cross-validations. The confusion matrix and the AUC of the ROC curves were calculated with a probability threshold of 0.5. Cohen’s kappa was utilized to assess the inter-rater reliability of the multivariate biomarker model by calculating the level of agreement between the predictive label and the actual label of patients. A Cohen’s kappa of above or equal to 0.7 is considered a substantial agreement.

### Other statistical analyses

To assess differences in patient characteristics and demographics, a Kruskal–Wallis test was employed for continuous variables, while Fisher’s exact test was applied to categorical variables. Statistical differences in the protein concentrations between disease groups were assessed using a one-way analysis of variance (ANOVA). If the difference was significant, multiple pairwise comparisons were performed with Bonferroni’s correction. Comparisons were adjusted for age and BMI.

## Supplementary Information

Additional file 1: Supplementary Tables. Table S1. Patient demographics and characteristics. Table S2. Tumor characteristics of endometrial cancer patients.

Additional file 2: Supplementary Figures. Figure S1. Protein concentrations in varying severities of EC compared to benign conditions. Figure S2. Univariate ROC curves of proteins discriminatory potential for EC types. Figure S3. Multivariate protein model of 11 proteins shows excellent discriminatory potential for EC. Figure S4. Protein concentrations positively correlate with tumor size and depth of myometrial invasion. Figure S5. Correlation of growth factors to other tested proteins. Figure S6. Angiopoietin-2, FAP, and VEGF-A positively correlate with each other to promote angiogenesis.

## Data Availability

The data supporting the conclusions of this article are included within the article and its Supplementary Information files. Additional data is available from the corresponding author upon reasonable request.

## Abbreviations

EC: Endometrial cancer
CVL: Cervicovaginal lavage
EEC: Endometrial endometrioid cancer
BMI: Body mass index
MMR: Mismatch repair
TVUS: Transvaginal ultrasound
AUC: Area under curve
ROC: Receiver operating characteristic
