# Supplementary material for: Cervicovaginal lavages uncover growth factors as key biomarkers for early diagnosis and prognosis of endometrial cancer
Source: Mol Biomed. 2024 Nov 8;5:55. doi: 10.1186/s43556-024-00219-6 (PMC11543965; doi:10.1186/s43556-024-00219-6)
Supplement: Supplementary file 1 — Additional file 1: Supplementary Tables. Table S1. Patient demographics and characteristics. Table S2. Tumor characteristics of endometrial cancer patients. [file 43556_2024_219_MOESM1_ESM.docx]

**Cervicovaginal Lavages Uncover Growth Factors as Key Biomarkers for Early Diagnosis and Prognosis of Endometrial Cancer.**

Hannah J Harris^1,2^, Paweł Łaniewski^3^, Haiyan Cui^4^, Denise J Roe^4,5^, Dana M Chase^6^, Melissa M Herbst-Kralovetz^1,3,4,*^

^1^ Department of Obstetrics and Gynecology, College of Medicine - Phoenix, University of Arizona, Phoenix, AZ, USA; ^2^ Department of Life Sciences, University of Bath, Bath, UK; ^3^ Department of Basic Medical Sciences, College of Medicine – Phoenix, University of Arizona, AZ, USA; ^4^ University of Arizona Cancer Center, Tucson, AZ, USA; ^5^ Department of Epidemiology and Biostatistics, Mel and Enid Zuckerman College of Public Health, University of Arizona, AZ, USA; ^6^ Division of Gynecologic Oncology, Department of Obstetrics and Gynecology, David Geffen School of Medicine at University of California Los Angeles, Los Angeles, CA, USA

*Correspondence: [mherbst1@arizona.edu](mailto:mherbst1@arizona.edu)

| **Supplementary Table 1.** Patient demographics and characteristics. Patient demographics and characteristics are separated based on disease groups: All (*n*=192), Benign conditions (*n*=108), endometrial hyperplasia (*n*=18) , grade 1/2 EEC (*n*=53), and other EC types (*n*=13). *P* values were calculated using the Kruskal-Wallis test for continuous variables and Fisher’s exact for categorical variables. Bonferroni's correction was applied if the overall difference was significant (p<0.05). Abbreviations: body mass index (BMI), endometrial cancer (EC), endometrial endometrioid carcinoma (EEC), general educational development (GED), medical history (hx), polycystic ovary syndrome (PCOS), standard deviation (SD). Groups: (1) benign conditions, (2) endometrial hyperplasia, (3) Grade 1/2 EEC, (4) other EMC subtypes. | | | | | | | | |
| --- | --- | --- | --- | --- | --- | --- | --- | --- |
|  | **All** | **Benign conditions** | **Endometrial hyperplasia** | **Grade 1/2 EEC** | **Other EC subtypes** | ***P* value** | | |
|  | (*n*=192) | (*n*=108) | (*n*=18) | (*n*=53) | (*n*=13) | **overall** | **paired** | |
| **Age (mean (SD))** (*n*=192) | 51.02 (12.45) | 45.55 (10.01) | 54.11 (13.35) | 58.73 (11.82) | 60.77 (8.06) | <0.0001 | (1) vs. (2) | 0.01 |
|  |  |  |  |  |  |  | (1) vs. (3) | <0.0001 |
|  |  |  |  |  |  |  | (1) vs. (4) | <0.0001 |
| **Race** (***n* (%))** (n=190) |  |  |  |  |  |  |  |  |
| White/Caucasian | 142 (74.74) | 78 (72.90) | 16 (88.89) | 37 (71.15) | 11 (84.62) | 0.004 | (1) vs. (3) | 0.045 |
| American Indian/Alaska Native | 15 (7.89) | 5 (4.67) | 1 (5.56) | 8 (15.38) | 1 (7.69) |  |  |  |
| Black/African American | 12 (6.32) | 11 (10.28) | 0 (0.00) | 1 (1.92) | 0 (0.00) |  |  |  |
| Mixed/Multi-racial | 9 (4.74) | 7 (6.54) | 0 (0.00) | 2 (3.95) | 0 (0.00) |  |  |  |
| Asian/Far East/South East | 4 (2.11) | 4 (3.74) | 0 (0.00) | 0 )0.00) | 0 (0.00) |  |  |  |
| Asian/Indian | 1 (0.53) | 0 (0.00) | 1 (5.56) | 0 (0.00) | 0 (0.00) |  |  |  |
| Native Hawaiian/Pacific Islander | 1 (0.53) | 0 (0.00) | 0 (0.00) | 1 (1.92) | 0 (0.00) |  |  |  |
| Middle Eastern/North African | 1 (0.53) | 0 (0.00) | 0 (0.00) | 0 (0.00) | 1 (7.69) |  |  |  |
| Not specified/other | 5 (2.63) | 2 (1.87) | 0 (0.00) | 3 (5.77) | 0 (0.00) |  |  |  |
| **Ethnicity (*n* (%))** (*n*=191) |  |  |  |  |  |  |  |  |
| Non-Hispanic | 141 (73.82) | 76 (70.37) | 14 (77.78) | 41 (78.85) | 10 (76.92) | 0.67 |  |  |
| Hispanic | 50 (26.18) | 32 (29.63) | 4 (22.22) | 11 (21.15) | 3 (23.08) |  |  |  |
| **BMI (*n* (%))** (*n*=192) |  |  |  |  |  |  |  |  |
| <25 | 29 (15.38) | 23 (21.30) | 0 (0.00) | 4 (7.55) | 2 (15.38) | <0.0001 | (1) vs. (2) | <0.0001 |
| 25-29 | 47 (24.38) | 38 (35.19) | 1 (5.56) | 6 (11.32) | 2 (15.38) |  | (1) vs. (3) | <0.0001 |
| 30-34 | 30 (15.63) | 19 (17.59) | 2 (11.11) | 6 (11.32) | 3 (23.08) |  |  |  |
| ≥35 | 86 (44.79) | 28 (25.93) | 15 (83.33) | 37 (69.81) | 6 (46.15) |  |  |  |
| **BMI (mean (SD))** (*n*=192) | 34.76(10.16) | 30.63 (7.54) | 41.49 (7.45) | 40.29 (11.07) | 37.22 (12.76) | <0.0001 | (1) vs. (2) | <0.0001 |
|  |  |  |  |  |  |  | (1) vs. (3) | <0.0001 |
|  |  |  |  |  |  |  | (2) vs. (3) | 0.005 |
| **Menopause status (*n* (%))** (*n*=190) |  |  |  |  |  |  |  |  |
| Premenopausal | 108 (56.84) | 89 (82.41) | 6 (33.33) | 12 (23.53) | 1 (7.69) | <0.0001 | (1) vs. (2) | <0.0001 |
| Postmenopausal | 82 (43.16) | 19 (17.59) | 12 (66.67) | 39 (76.47) | 12 (92.31) |  | (1) vs. (3) | <0.0001 |
|  |  |  |  |  |  |  | (1) vs. (4) | <0.0001 |
| **Education (*n* (%))** (*n*=175) |  |  |  |  |  |  |  |  |
| Less than high school | 10 ((5.71) | 3 (2.86) | 2 (14.29) | 4 (8.89) | 1 (9.09) | 0.36 |  |  |
| High school diploma or GED | 38 (21.71) | 20 (19.05) | 6 (42.86) | 10 (22.22) | 2 (18.18) |  |  |  |
| Some college | 42 (24.00) | 24 (22.86) | 4 (28.57) | 12 (26.67) | 2 (18.18) |  |  |  |
| Association degree or technical certification | 35 (20.00) | 22 (20.95) | 1 (7.14) | 9 (20.00) | 3 (27.27) |  |  |  |
| Bachelor’s degree | 30 (17.14) | 22 (20.95) | 0 (0.00) | 5 (11.11) | 3 (27.27) |  |  |  |
| Master’s/doctor’s degree | 20 (11.43) | 14 (13.33) | 1 (7.14) | 5 (11.11) | 0 (0.00) |  |  |  |
| **Income (*n* (%))** (*n*=168) |  |  |  |  |  |  |  |  |
| <10,000 | 8 (4.60) | 3 (3.03) | 0 (0.00) | 5 (11.36) | 0 (0.00) | 0.002 | (1) vs. (3) | 0.002 |
| 10,000-25,000 | 26 (14.94) | 10 (10.10) | 6 (42.86) | 7 (15.91) | 3 (27.27) |  |  |  |
| 25,000-50,000 | 39 (22.41) | 13 (13.13) | 3 (21.43) | 16 (36.36) | 4 (36.36) |  |  |  |
| 50,000-75,000 | 32 (18.39) | 23 (23.23) | 2 (14.29) | 6 (13.64) | 1 (9.09) |  |  |  |
| 75,000-100,000 | 18 (10.34) | 15 (15.15) | 1 (7.14) | 1 (2.27) | 1 (9.09) |  |  |  |
| >100,000 | 32 (18.39) | 24 (24.24) | 1 (7.14) | 3 (6.82) | 2 (18.18) |  |  |  |
| Don’t know/refused | 19 (10.32) | 11 (11.11) | 1 (7.14) | 6 (13.64) | 0 (0.00) |  |  |  |
| **Marital status (*n* (%))** (*n*=191) |  |  |  |  |  |  |  |  |
| Single/divorced/ widowed | 85 (44.50) | 41 (37.96) | 8 (44.44) | 30 (57.69) | 6 (46.15) | 0.41 |  |  |
| Married | 96 (50.26) | 60 (55.56) | 8 (44.44) | 21 (40.38) | 7 (53.85) |  |  |  |
| Cohabitating | 6 (3.14) | 5 (4.63) | 1 (5.56) | 0 (0.00) | 0 (0.00) |  |  |  |
| Other | 4 (2.09) | 2 (11.85) | 1 (5.56) | 1 (1.54) | 0 (0.00) |  |  |  |
| **Sexual orientation (*n* (%))** (*n*=171) |  |  |  |  |  |  |  |  |
| Heterosexual | 164 (95.91 | 93 (93.00) | 14 (100.00) | 44 (10.00) | 13 (100.00) | 0.52 |  |  |
| Bisexual | 2 (1.17) | 2 (2.00) | 0 (0.00) | 0 (0.00) | 0 (0.00) |  |  |  |
| Homosexual | 5 (2.92) | 5 (5.00) | 0 (0.00) | 0 (0.00) | 0 (0.00) |  |  |  |
| **Employment status (*n* (%))** (*n*=174) |  |  |  |  |  |  |  |  |
| Employed | 100 (57.47) | 75 (72.82) | 7 (50.00) | 14 (31.11) | 4 (33.33) | <0.0001 | (1) vs. (3) | <0.0001 |
| Unemployed | 74 (42.53)) | 28 (27.18) | 7 (50.00) | 31 (68.89) | 8 (66.67) |  | (1) vs. (4) | 0.04 |
| **Contraceptive use in past 6 months (*n* (%))** |  |  |  |  |  |  |  |  |
| Birth control pill (*n*=127) |  |  |  |  |  |  |  |  |
| Yes | 15 (11.81) | 12 (16.00) | 0 (0.00) | 3 (9.38) | 0 (0.00) | 0.25 |  |  |
| No | 112 (88.19) | 63 (84.00) | 11 (100.00) | 29 (90.63) | 9 (100.00) |  |  |  |
| Depo-Provera® (*n*=74) |  |  |  |  |  |  |  |  |
| Yes | 1 (1.35) | 0 (0.00) | 0 (0.00) | 0 (0.00) | 1 (20.00) | 0.07 |  |  |
| No | 73 (98.65) | 45 (100.00) | 9 (100.00) | 15 (100.00) | 4 (80.00) |  |  |  |
| Paragard® (*n*=64) |  |  |  |  |  |  |  |  |
| Yes | 2 (3.13) | 1 (2.50) | 0 (0.00) | 1 (7.14) | 0 (0.00) | 0.61 |  |  |
| No | 62 (96.88) | 39 (97.50 | 6 (100.00) | 13 (92.86) | 4 (100.00) |  |  |  |
| Hormone IUD (*n*=68) |  |  |  |  |  |  |  |  |
| Yes | 10 (14.71) | 9 (20.43) | 0 (0.00) | 0 (0.00) | 1 (20.00) | 0.13 |  |  |
| No | 58 (85.29) | 33 (78.57) | 7 (100.00) | 14 (100.00) | 4 (80.00) |  |  |  |
| **Type of contraceptives (*n* (%))** |  |  |  |  |  |  |  |  |
| Hormonal (*n*=138) |  |  |  |  |  |  |  |  |
| Yes | 36 (26.09) | 26 (32.10) | 0 (0.00) | 5 (14.71) | 5 (45.45) | 0.01 | (2) vs. (4) | 0.05 |
| No | 102 (73.91) | 55 (67.90) | 12 (100.00) | 29 (85.29) | 6 (54.55) |  |  |  |
|  |  |  |  |  |  |  |  |  |
| Non-hormonal (*n*=64) |  |  |  |  |  |  |  |  |
| Yes | 2 (3.13) | 1 (2.50) | 0 (0.00) | 1 (7.14) | 0 (0.00) | 0.62 |  |  |
| No | 62 (96.88) | 39 (97.50 | 6 (100.00) | 13 (92.86) | 4 (100.00) |  |  |  |
| **Surgical contraception (*n* (%))** (*n*=100) |  |  |  |  |  |  |  |  |
| Tubal ligation | 40 (40.00) | 25 (40.98) | 3 (37.50) | 10 (41.67) | 2 (20.00) | 0.73 |  |  |
| Essure® | 5 (5.00) | 5 (8.20) | 0 (0.00) | 0 (0.00) | 0 (0.00) |  |  |  |
| Both ovaries removed | 1 1.00) | 1 (1.64) | 0 (0.00) | 0 (0.00) | 0 (0.00) |  |  |  |
| Tubes and ovaries removed | 3 (3.00) | 2 (3.28) | 1 (12.50) | 0 (0.00) | 0 (0.0) |  |  |  |
| None | 51 (51.00) | 19 (61.29) | 4 (50.00) | 14 (58.33) | 8 (80.00) |  |  |  |
| **Hormone replacement therapy (*n* (%))** |  |  |  |  |  |  |  |  |
| Hormone receptor modulator, agonist or antagonist (*n*=64) |  |  |  |  |  |  |  |  |
| Yes | 2 (3.13) | 2 (5.13) | 0 (0.00) | 0 (0.00) | 0 (0.00) | 0.99 |  |  |
| No | 62 (96.88) | 37 (94.87) | 6 (100.00) | 14 (100.00) | 5 (100.00) |  |  |  |
| Hormone treatment (estrogen, estrogen/progesterone) (*n*=80) |  |  |  |  |  |  |  |  |
| Yes | 15 (18.75) | 10 (20.41) | 0 (0.00) | 2 (11.11) | 3 (42.86) | 0.11 |  |  |
| No | 65 (81.25)) | 39 (79.59) | 7 (100.00) | 16 (88.89) | 4 (57.14) |  |  |  |
| **Parity (*n* (%))** (*n*=191) |  |  |  |  |  |  |  |  |
| 0 | 44 (23.04) | 22 (20.56) | 4 (22.22) | 15 (28.30) | 3 (23.08) | 0.3 |  |  |
| 1 | 24 (12.57) | 9 (8.41) | 4 (22.22) | 8 (15.09) | 3 (23.08) |  |  |  |
| 2 | 42 (21.99) | 23 (21.50) | 7 (38.89) | 10 (18.87) | 2 (15.38) |  |  |  |
| 3 | 44 (23.04) | 27 (25.23) | 2 (11.11) | 13 (24.53) | 2 (15.38) |  |  |  |
| 4+ | 37 (19.37) | 26 (24.30) | 1 (5.56) | 7 (13.21) | 3 (23.08) |  |  |  |
| **Heavy periods (*n* (%))** (*n*=150) |  |  |  |  |  |  |  |  |
| Light | 9 (6.00) | 5 (5.32) | 0 (0.00) | 2 (5.88) | 2 (20.00) | 0.01 | (1) vs. (3) | 0.04 |
| Moderate | 48 (32.00) | 21 (22.34) | 6 (50.00) | 17 (50.0) | 4 (40.00) |  |  |  |
| Heavy | 93 (62.00) | 68 (72.34) | 6 (50.00) | 15 (44.12) | 4 (40.00) |  |  |  |
| **Douching (*n* (%))** (*n*=161) |  |  |  |  |  |  |  |  |
| Yes | 26 (16.15) | 15 (15.96) | 3 (21.43) | 7 (16.28) | 1 (10.00) | 0.9 |  |  |
| No | 135 (83.85) | 79 (84.04) | 11 (78.57) | 36 (83.72) | 9 (90.00) |  |  |  |
| **Dilation and curettage (*n* (%))** (*n*=192) |  |  |  |  |  |  |  |  |
| Yes | 58 (30.21) | 19 (17.59) | 7 (38.89) | 25 (47.17) | 7 (53.85) | 0.0002 | (1) vs (3) | 0.0006 |
| No | 134 (69.79) | 89 (82.41) | 11 (61.11) | 28 (52.83) | 6 (46.15) |  | (1) vs (4) | 0.04 |
| **Uterine manipulator type used (*n* (%))** (*n*=185) |  |  |  |  |  |  |  |  |
| Fornisee® | 63 (34.05) | 61 (58.65) | 2 (12.50) | 0 (0.00) | 0 (0.00) | <0.0001 | (1) vs. (2) | <0.0001 |
| Sacrocervicopexy | 0 (0.00) | 0 (0.00) | 0 (0.00) | 0 (0.00) | 0 (0.00) |  | (1) vs. (3) | <0.0001 |
| Delineator™ | 77 (41.62) | 31 (29.81) | 4 (25.00) | 36 (67.92) | 6 (50.00) |  | (1) vs. (4) | <0.0001 |
| VCare® | 21 (11.35) | 5 (4.81) | 4 (25.00) | 7 (13.21) | 5 (41.67)) |  | (2) vs. (3) | 0.01 |
| RUMI® | 23 (12.43) | 6 (5.77) | 6 (37.50) | 10 (18.87) | 1 (8.33) |  |  |  |
| Sponge stick | 1 (0.54) | 1 (0.96) | 0 (0.00) | 0 (0.00) | 0 (0.00) |  |  |  |
| **Chronic pelvic pain hx (*n* (%))** (*n*=155) |  |  |  |  |  |  |  |  |
| Yes | 64 (41.29) | 50 (54.95) | 2 (15.38) | 10 (25.00) | 2 (18.18) | 0.001 | (1) vs. (3) | 0.01 |
| No | 91 (58.71) | 41 (45.05) | 11 (84.62) | 30 (75.00) | 9 (81.82) |  |  |  |
|  |  |  |  |  |  |  |  |  |
| **Endometriosis hx (*n* (%))** (*n*=162) |  |  |  |  |  |  |  |  |
| Yes | 43 (26.54) | 28 (29.47) | 2 (14.29) | 12 (28.57) | 1 (9.09) | 0.35 |  |  |
| No | 119 (73.46) | 67 (70.53) | 12 (85.71) | 30 (71.43) | 10 (90.91) |  |  |  |
| **PCOS hx (*n* (%))** (*n*=154) |  |  |  |  |  |  |  |  |
| Yes | 16 (10.39) | 10 (11.36) | 2 (13.33) | 4 (10.00) | 0 (0.00 | 0.68 |  |  |
| No | 138 (89.61) | 78 (88.64) | 13 (86.67) | 36 (90.00) | 11 (100.00) |  |  |  |
| **Diabetes (*n* (%))** (*n*=192) |  |  |  |  |  |  |  |  |
| Yes | 48 (25.00) | 22 (20.37) | 5 (27.78) | 21 (39.62) | 0 (0.00) | 0.006 | (3) vs. (4) | 0.03 |
| No | 144 (75.00) | 86 (79.63) | 13 (72.22) | 32 (60.38) | 13 (100.00) |  |  |  |
|  |  |  |  |  |  |  |  |  |
| **Hypertension (*n* (%))** (*n*=192) |  |  |  |  |  |  |  |  |
| Yes | 65 (33.85) | 25 (23.15) | 6 (33.33) | 27 (50.94) | 7 (53.85) | 0.002 | (1) vs. (3) | 0.001 |
| No | 127 (66.15) | 83 (76.85) | 12 (66.67) | 26 (49.06) | 6 (46.15) |  |  |  |
| **Antibiotics (recent use) (*n* (%))** (*n*=161) |  |  |  |  |  |  |  |  |
| Yes | 41 (25.47) | 23 (24.21) | 4 (26.67) | 10 (24.39) | 4 (40.00) | 0.75 |  |  |
| No | 120 (74.53) | 72 (75.79) | 11 (73.33) | 31 (75.16) | 6 (60.00) |  |  |  |
| **Alcohol use (*n* (%))** (*n*=175) |  |  |  |  |  |  |  |  |
| Yes | 72 (41.41) | 50 (49.50) | 7 (43.75) | 11 (24.44) | 4 (30.77) | 0.11 |  |  |
| No | 94 (69.23) | 47 (46.53) | 8 (50.00) | 30 (66.67) | 9 (69.23) |  |  |  |
| Quit | 9 (5.14) | 4 (3.96) | 1 (6.25) | 4 (8.89) | 0 (0.00) |  |  |  |
| **Tobacco use (*n* (%))** (*n*=184) |  |  |  |  |  |  |  |  |
| Yes | 19 (10.33) | 14 (13.46) | 1 (5.56) | 3 (6.12) | 1 (7.69) | 0.15 |  |  |
| No | 55 (29.89) | 34 (32.69) | 4 (22.22) | 13 (26.53) | 4 (30.77) |  |  |  |
| Never | 89 (48.37) | 47 (45.19) | 12 (66.67) | 22 (44.90) | 8 (61.54) |  |  |  |
| Quit | 21 (11.41) | 9 (8.65) | 1 (5.56) | 11 (22.45) | 0 (0.00) |  |  |  |

|  | *n* (%) |
| --- | --- |
| Tumor Characteristic: |  |
|  |  |
| **Histological type (*n*=66)** |  |
| Endometrioid adenocarcinoma | 33 (50) |
| Endometrioid carcinoma | 26 (39.4) |
| Serous carcinoma | 4 (6.1) |
| Other | 3 (4.5) |
| **FIGO stage (*n*=62)** |  |
| I | 1 (1.6) |
| IA | 43 (69.4) |
| IB | 10 (16.1) |
| II | 2 (3.2) |
| IIIC | 4 (6.5) |
| IV | 2 (3.2) |
| **Tumor grade (*n*=66)** |  |
| 1 | 42 (63.6) |
| 2 | 15 (22.7) |
| 3 | 9 (13.6) |
| **Tumor size (*n*=62)** |  |
| ≤2cm | 17 (27.4) |
| >2cm | 45 (72.6) |
| **Presence of myometrial invasion (*n*=66)** |  |
| no | 20 (30.3) |
| yes | 46 (60.6) |
| **Lymphovascular invasion (*n*=65)** |  |
| no | 61 (93.8) |
| yes | 4 (6.2) |
| **MMR status (*n*=60)** |  |
| MMR-deficient | 14 (23.3) |
| MMR-proficient | 46 (76.7) |

**Supplementary Table 2.** Tumor characteristics of endometrial cancer patients. Data from pathology reports on EC tumor characteristics such as, histological type, FIGO stage, tumor grade, tumor size, myometrial invasion, lymphovascular invasion and MMR status are summarized below. Data availability of each characteristic is indicated in the table.
