# Supplementary material for: Cervicovaginal lavages uncover growth factors as key biomarkers for early diagnosis and prognosis of endometrial cancer
Source: Mol Biomed. 2024 Nov 8;5:55. doi: 10.1186/s43556-024-00219-6 (PMC11543965; doi:10.1186/s43556-024-00219-6)
Supplement: Supplementary file 2 — Additional file 2: Supplementary Figures. Figure S1. Protein concentrations in varying severities of EC compared to benign conditions. Figure S2. Univariate ROC curves of proteins discriminatory potential for EC types. Figure S3. Multivariate protein model of 11 proteins shows excellent discriminatory potential for EC. Figure S4. Protein concentrations positively correlate with tumor size and depth of myometrial invasion. Figure S5. Correlation of growth factors to other tested proteins. Figure S6. Angiopoietin-2, FAP, and VEGF-A positively correlate with each other to promote angiogenesis. [file 43556_2024_219_MOESM2_ESM.pdf]

Cervicovaginal Lavages Uncover Growth Factors as Key Biomarkers Early Diagnosis and Prognosis of Endometrial Cancer.

Hannah J Harris[1,2], Paweł Łaniewski[3], Haiyan Cui[4], Denise J Roe[4,5], Dana M Chase[6], Melissa M Herbst-Kralovetz[1,3,4,*]

[1] Department of Obstetrics and Gynecology, College of Medicine - Phoenix, University of Arizona, Phoenix, AZ, USA; [2] Department of Life Sciences, University of Bath, Bath, UK; [3] Department of Basic Medical Sciences, College of Medicine – Phoenix, University of Arizona, AZ, USA; [4] University of Arizona Cancer Center, Tucson, AZ, USA; [5] Department of Epidemiology and Biostatistics, Mel and Enid Zuckerman College of Public Health, University of Arizona, AZ, USA; [6] Division of Gynecologic Oncology, Department of Obstetrics and Gynecology, David Geffen School of Medicine at University of California Los Angeles, Los Angeles, CA, USA

*Correspondence: mherbst1@arizona.edu

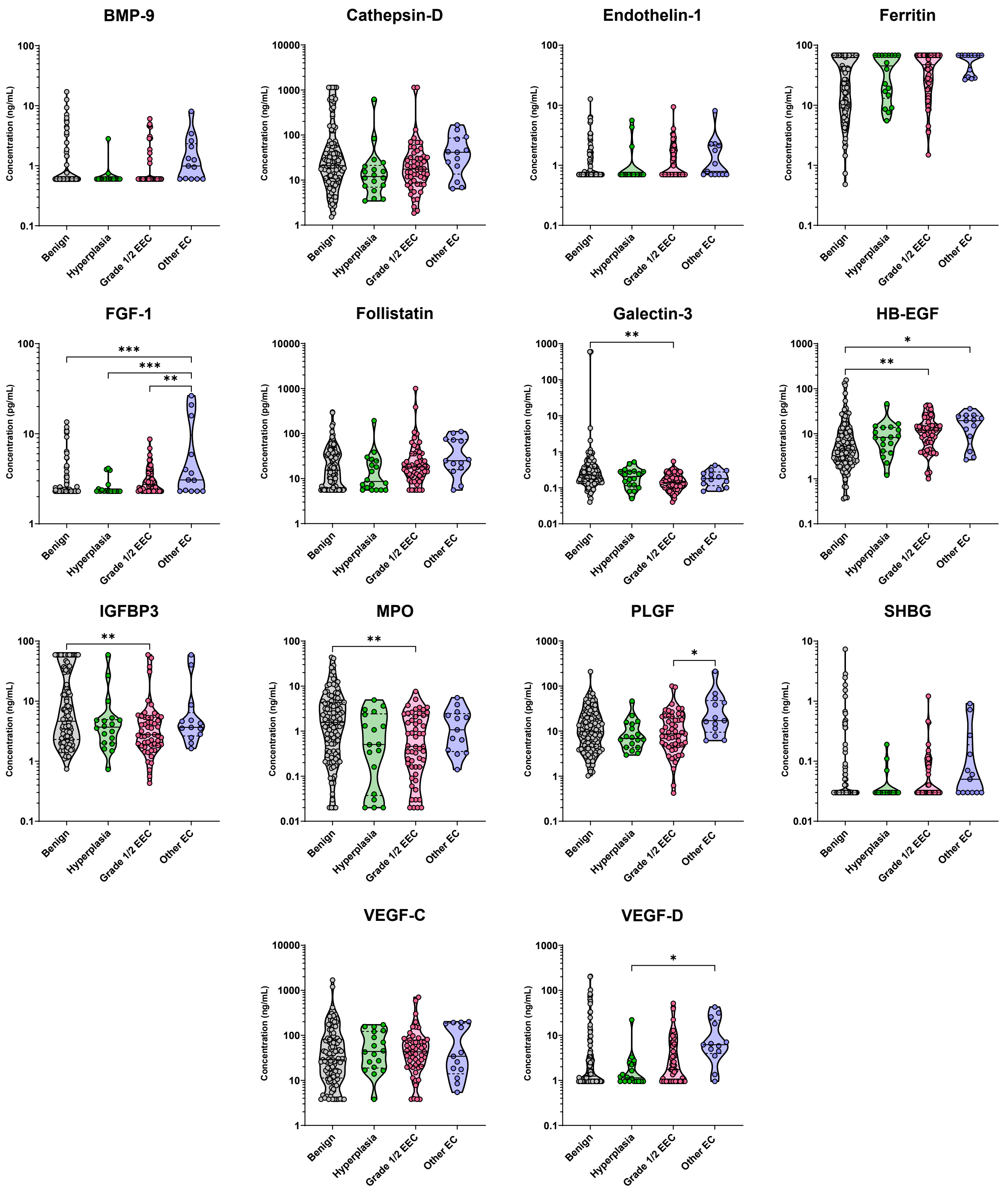

Figure S1. Protein concentrations in varying severities of EC compared to benign conditions. Truncated violin plots depict the concentration of 14 proteins across all the disease groups (Benign, hyperplasia, grade 1/2 EEC, and other EC types). The solid horizontal line represents the median value. P values were calculated using a one-way ANOVA with Bonferroni's correction. Asterisks indicate the p values (* $p<0.05$, ** $p<0.01$, *** $p<0.001$).

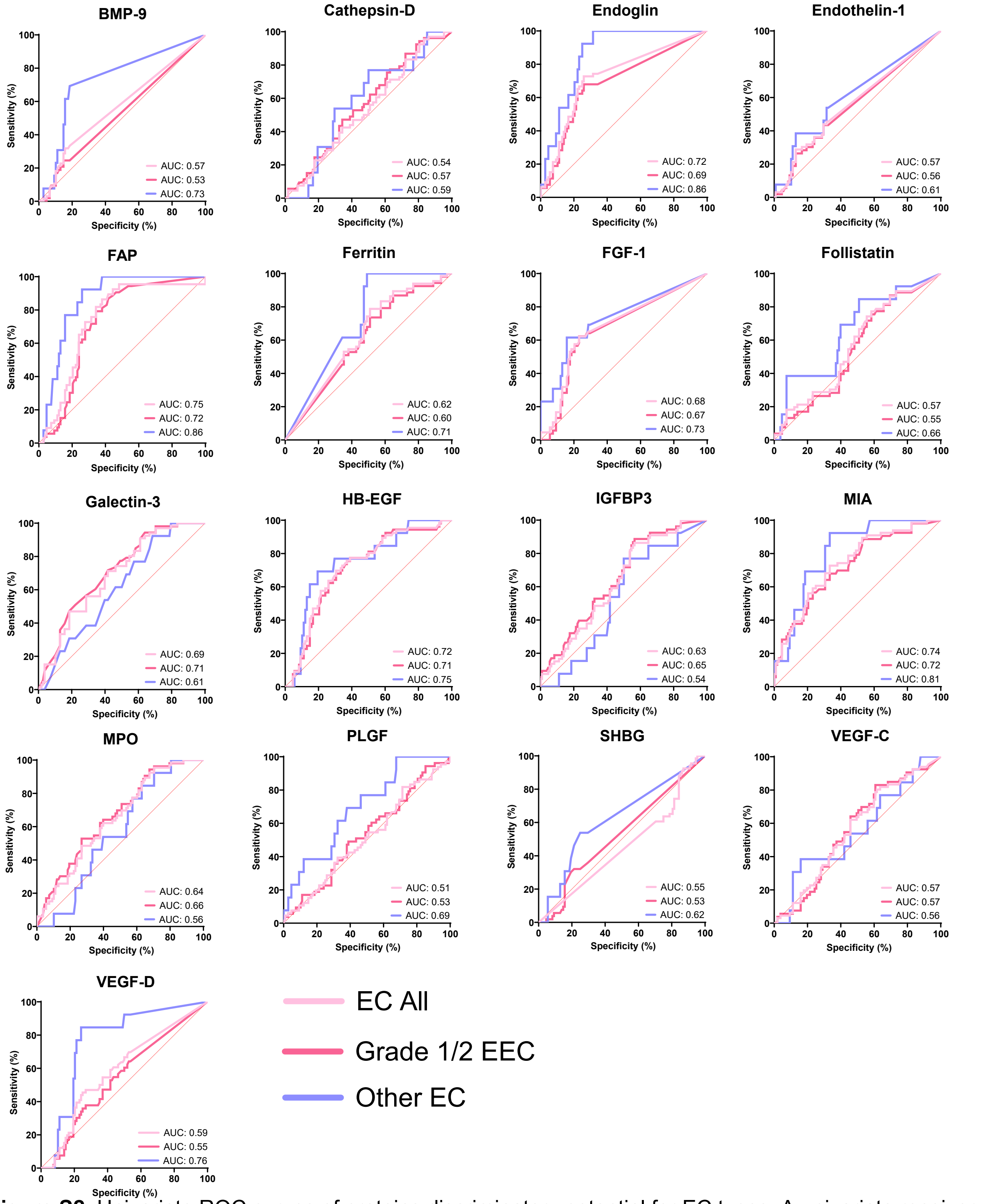

Figure S2. Univariate ROC curves of proteins discriminatory potential for EC types. A univariate receiver operating characteristics (ROC) analysis was used to assess the potential for individual proteins to discriminate EC types (EC all, Grade 1/2 EEC, and Other EC types) from benign conditions. The area under the curve (AUC) was calculated for each protein. Proteins with an AUC above 0.8 were considered good discriminatory biomarkers. Three proteins (endoglin, FAP, and MIA) displayed good discriminatory potential for other EC types.

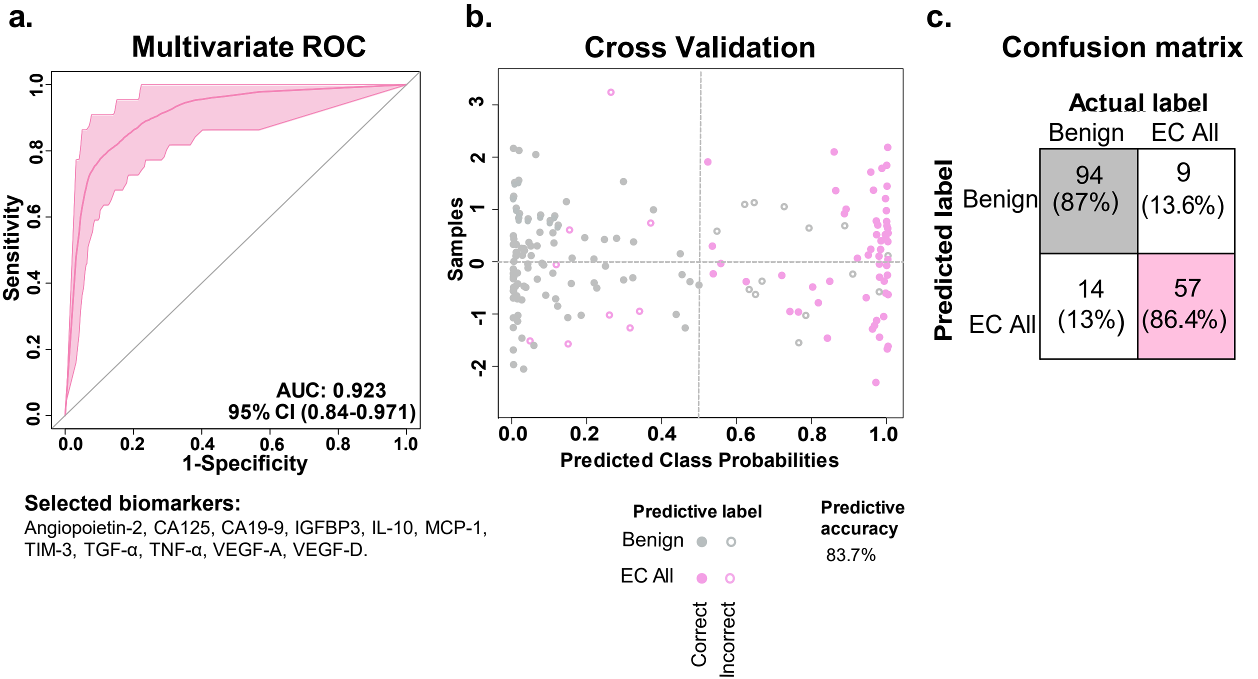

Figure S3. Multivariate protein model of 11 proteins shows excellent discriminatory potential for EC. **a** Multivariate ROC curve of 11 proteins displays excellent discriminatory potential for EC with an area under the curve (AUC) of 0.923. Proteins were selected based on high least absolute shrinkage and selection operator (LASSO) frequencies and empirical testing of combinations of proteins. A Monte Carlo cross-validation (MCCV) **b** was used to test the predictive accuracy of the model, which was found to be 83.7%. The predicted class probabilities of the samples are shown using the classifier at a threshold of 0.5. **c** The confusion matrix depicts the number of patients this protein model accurately classified to their respective disease groups. This multivariate model accurately classified 151/174 samples with a specificity of 87% and a sensitivity of 86.4%.

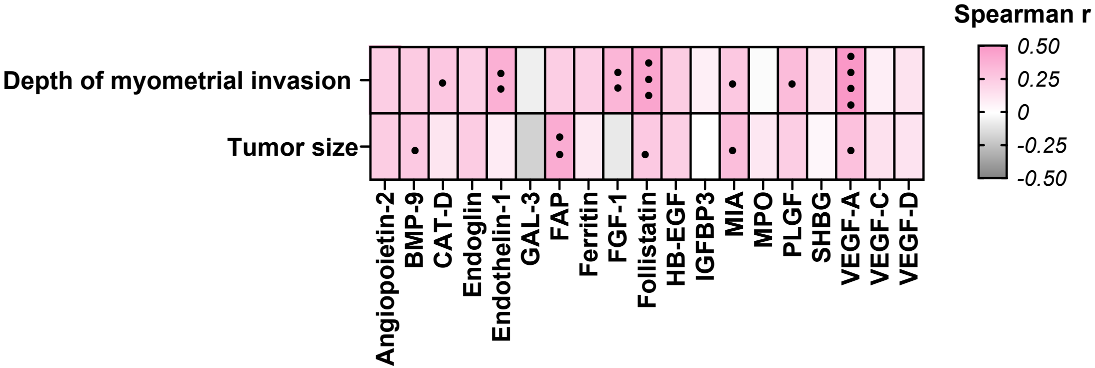

Figure S4. Protein concentrations positively correlate with tumor size and depth of myometrial invasion. Spearman rank correlation coefficients were calculated between protein concentrations and depth of myometrial invasion (measured in mm) and tumor size (measured in cm). These values are represented in a heatmap. P values are represented with black circles * p<0.05, ** p<0.01, *** p<0.001, **** p<0.0001.

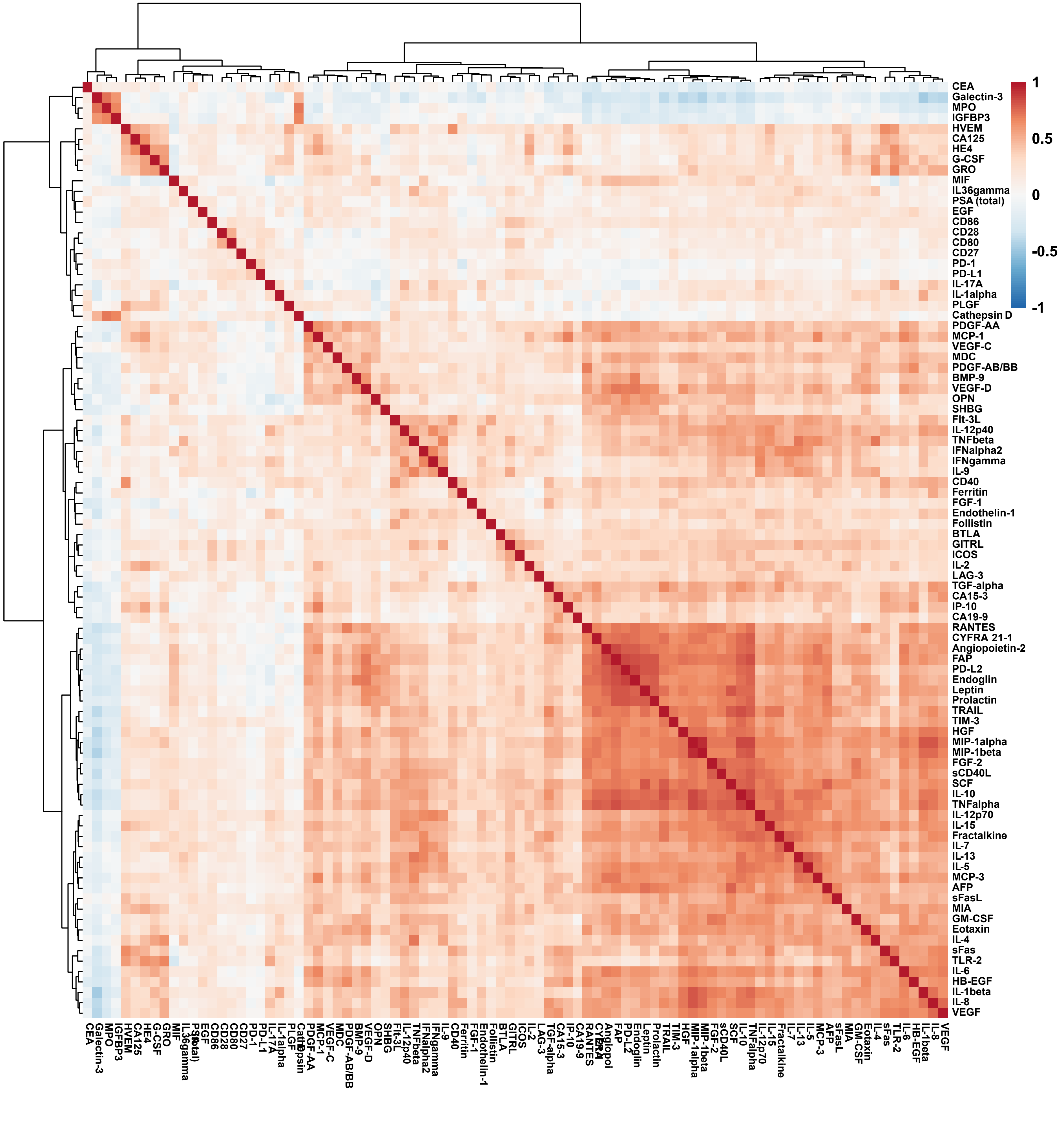

Figure S5. Correlation of pro- and anti-inflammatory cytokines, chemokines, and growth factors. Heatmap depicts correlation coefficients of 90 proteins with one another. Spearman rank correlation analysis was utilized to calculate correlation coefficients. Hierarchical cluster analysis was conducted, data was not centered or scaled. Euclidean distance and Ward linkage method were used for clustering.

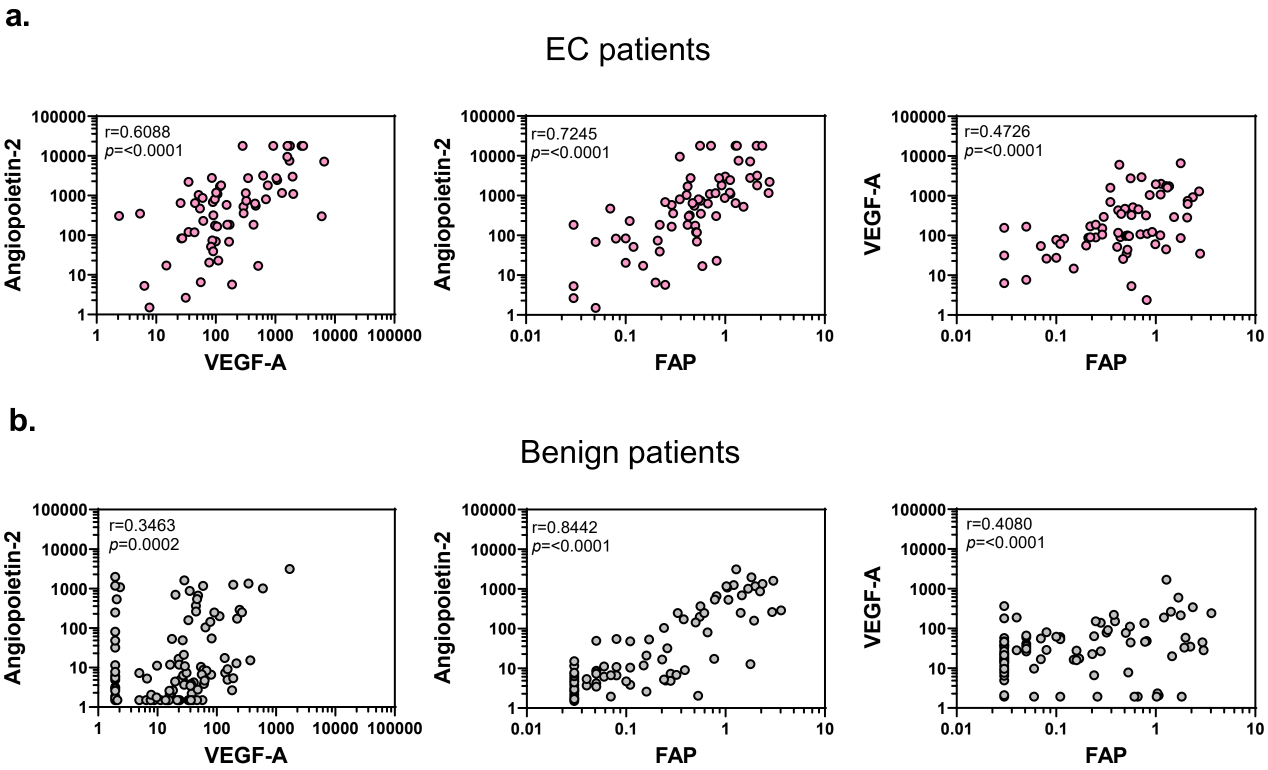

Figure S6. Angiopoietin-2, FAP, and VEGF-A positively correlate with each other to promote angiogenesis. Spearman rank correlation analysis revealed that Angiopoietin-2 positively correlates with VEGF-A and FAP, also VEGF-A positively correlates with FAP in the EC patients **a**. Spearman rank correlation analysis was also conducted on the levels of angiopoietin-2, FAP, and VEGF-A in the benign patients **b**.
